# Diversity of pathogenic *Pseudomonas* isolated from citrus in Tunisia

**DOI:** 10.1186/s13568-020-01134-z

**Published:** 2020-11-01

**Authors:** Maroua Oueslati, Magdalena Mulet, Mohamed Zouaoui, Charlotte Chandeysson, Jorge Lalucat, Mohamed Rabeh Hajlaoui, Odile Berge, Elena García-Valdés, Najla Sadfi-Zouaoui

**Affiliations:** 1 Laboratoire de Mycologie, Pathologies et Biomarqueurs (LR16ES05), Département de Biologie, Université de Tunis-El Manar, 2092 Tunis, Tunisie; 2 Microbiologia, Departament de Biologia, Edifici Guillem Colom, Universitat de Les Illes Balears, Campus UIB, 07122 Palma de Mallorca, Spain; 3 INRAE, Pathologie Végétale, 84140 Monfavet, France; 4 Institut Mediterrani D’Estudis Avançats (IMEDEA, CSIC-UIB), Campus UIB, 07122 Palma de Mallorca, Spain; 5 Laboratoire de Biotechnologie Appliquée à l’Agriculture, INRA Tunisia, Université de Carthage, 2094 Ariana, Tunisia

**Keywords:** Citrus, Cultivars, *Pseudomonas syringae*, *rpoD*, Pathogenicity

## Abstract

The damages observed in Tunisian citrus orchards have prompted studies on the *Pseudomonas* spp*.* responsible for blast and black pit. Prospective orchards between 2015 and 2017 showed that the diseases rapidly spread geographically and to new cultivars. A screening of *Pseudomonas* spp. isolated from symptomatic trees revealed their wide diversity according to phylogenetic analysis of their housekeeping *rpoD* and *cts* genes. The majority of strains were affiliated to *Pseudomonas syringae* pv. *syringae* (Phylogroup PG02b), previously described in Tunisia. However, they exhibited various BOX-PCR fingerprints and were not clonal. This work demonstrated, for the first time in Tunisia, the involvement of *Pseudomonas cerasi* (PG02a) and *Pseudomonas congelans* (PG02c). The latter did not show significant pathogenicity on citrus, but was pathogenic on cantaloupe and active for ice nucleation that could play a role in the disease. A comparative phylogenetic study of citrus pathogens from Iran, Montenegro and Tunisia revealed that *P. syringae* (PG02b) strains are closely related but again not clonal. Interestingly *P. cerasi* (PG02a) was isolated in two countries and seems to outspread. However, its role in the diseases is not fully understood and it should be monitored in future studies. The diversity of pathogenic *Pseudomonas* spp. and the extension of the diseases highlight that they have become complex and synergistic. It opens questions about which factors favor diseases and how to fight against them efficiently and with sustainable means.

## Key points

- Citrus blast and citrus black pit were spread to new areas and cultivars in Tunisia.
- In addition to *Pseudomonas syringae* (Phylogroup PG02b), *Pseudomonas congelans* (PG02c) and *Pseudomonas cerasi* (PG02a) are now involved in the disease.
- A group of closely related strains is responsible for the disease in Tunisia, Montenegro and Iran.

## Introduction

*Pseudomonas syringae* has a huge impact on our scientific understanding of microbial pathogenicity, and continues to cause economically important plant diseases to both woody and annual crops (Lamichhane et al. 2015). It represents not only the first plant pathogenic bacteria but also the top of all time pathogen charts including fungi and oomycetes (Mansfield et al. 2012). Furthermore, in three consecutive years (January 2015 to July 2018), reports of diseases caused by strains of the *P. syringae* species group are more frequent than those caused by any other group of phytopathogenic bacteria (Morris et al. 2019). The wide genetic diversity of *P. syringae* pv. *syringae* is probably the cause of its large host range (Martín- Sanz et al. 2013). Many studies have demonstrated the transmissible nature of *P. syringae*, but it is also a remarkably adaptive pathogen, isolated from non-agricultural sites, in astonishing substrates such as snowmelt (Monteil et al. 2011) or epilithic biofilms (Morris et al. 2007). In this context, *P. syringae* has been proposed as a potential contributing factor in the formation of rain and snowfall, shaping the water cycle on Earth due to its efficiency as ice nucleators (Morris et al. 2013; Lamichhane et al. 2014). In agriculture, the ice nucleation activity of *P. syringae* can influence the transition from the epiphytic to the endophytic phase as the damage of frost leads to the creation of openings on the surface of plants to facilitate the entry of bacteria (Xin et al. 2018). *P. syringae* pv. *syringae* specially is a potent epiphytic bacterium and under favorable environmental conditions, the bacterial population colonizing the plant can be good predictors of later endophytic populations and disease outbreaks (Hirano and Upper 2000). On citrus orchards, in the Mediterranean countries *P. syringae* is responsible for two damaging diseases including black pit and blast (Ivanović et al. 2017; Abdellatif et al. 2017). Only few studies were developed taking this problem into account. Citrus blast has been reported in Turkey (Mirik et al. 2005), Iran (Beiki et al. 2016) and Montenegro (Ivanović et al. 2017). In Tunisia, *P. syringae* has been described on tomato (Mensi et al. 2018), on citrus (Abdellatif et al. 2015) and it was isolated from weeds and plant debris that can be the source of inoculums to trigger citrus blast and black pit diseases (Mougou and Boughalleb-M’Hamdi 2016). Given the significant damages observed in the field, the quick emergence of the disease and the economic importance of citrus cultivation in Tunisia, research on these diseases has recently grown. So far, the polyphagous nature and the diversity of these bacteria as well as its adaptive and resistant capacities have been more or less under estimated in Tunisia, although several studies focused on citrus blast and citrus black pit. Only similar pathogenic *P. syringae* strains belonging to phylogroup PG02b as described by Berge et al. (2014) were described (Mougou and Boughalleb-M'hamdi 2016; Abdellatif et al. 2017). In Montenegro, strains isolated from necrotic mandarin buds have been identified as one homogenous lineage of *P. syringae* pv. *syringae* (Ivanović et al. 2017). On the contrary, a more open approach was used in the citrus growing provinces of northern Iran where Beiki et al. (2016) revealed that several *Pseudomonas* species are involved in citrus blast. The disease was caused by pathogenic *P. viridiflava*, *P. syringae* and by *P. lurida*, *P. orientalis*, *P. simiae* and *P. moraviensis* reported for the first time in the disease (Beiki et al. 2016). In the same region, a new pathogenic species from the *P. syringae* group, *P. caspiana* was isolated from citrus leaf and stems symptoms (Busquets et al. 2017). It seems that many *Pseudomonas* species could be involved in citrus blast and black pit and that these diseases are more complex than previously thought. In order to better understand the situation in Tunisian orchards, Oueslati et al. (2019), using a similar approach, identified two new pathogenic *Pseudomonas* species involved in citrus black pit disease in different Tunisian sites: *P. kairouanensis* and *P. nabeulensis*. In this study, we aimed to explore the diversity and population structure of the pathogenic bacteria from the *P. syringae* group on citrus in Tunisia. A large survey of main citrus production regions during three seasons allowed the isolation of 820 strains from citrus fruits, branches and leaves with black pit, gummosis and blast diseases. Several species of the *P. syringae* group were identified with a chemotaxonomic approach and confirmed by a phylogenetic analysis of their *cts* or *rpoD* gene sequences two appropriate biomarkers for *Pseudomonas* spp. (Mulet et al. 2010; Sánchez et al. 2014a; Berge et al. 2014). They were characterized for some phenotypic and pathogenic traits and their genotypic diversity was studied. This study showed clearly for the first time, the spread of the disease to the Kairouan region, and that new cultivars (cvs) such as *Citrus limon* cv. ‘Lunari’, *C. reticulata* cv. ‘Hernandina’ and *C. sinensis* cvs. ‘Maltaise’ and ‘Valencia Late’ are now affected by the diseases. In order to control these diseases, it will be necessary to better describe and understand the presence of the bacterial population involved and the role of their diversity.

## Materials and methods

### Sampling strategy

**Fig.1:**
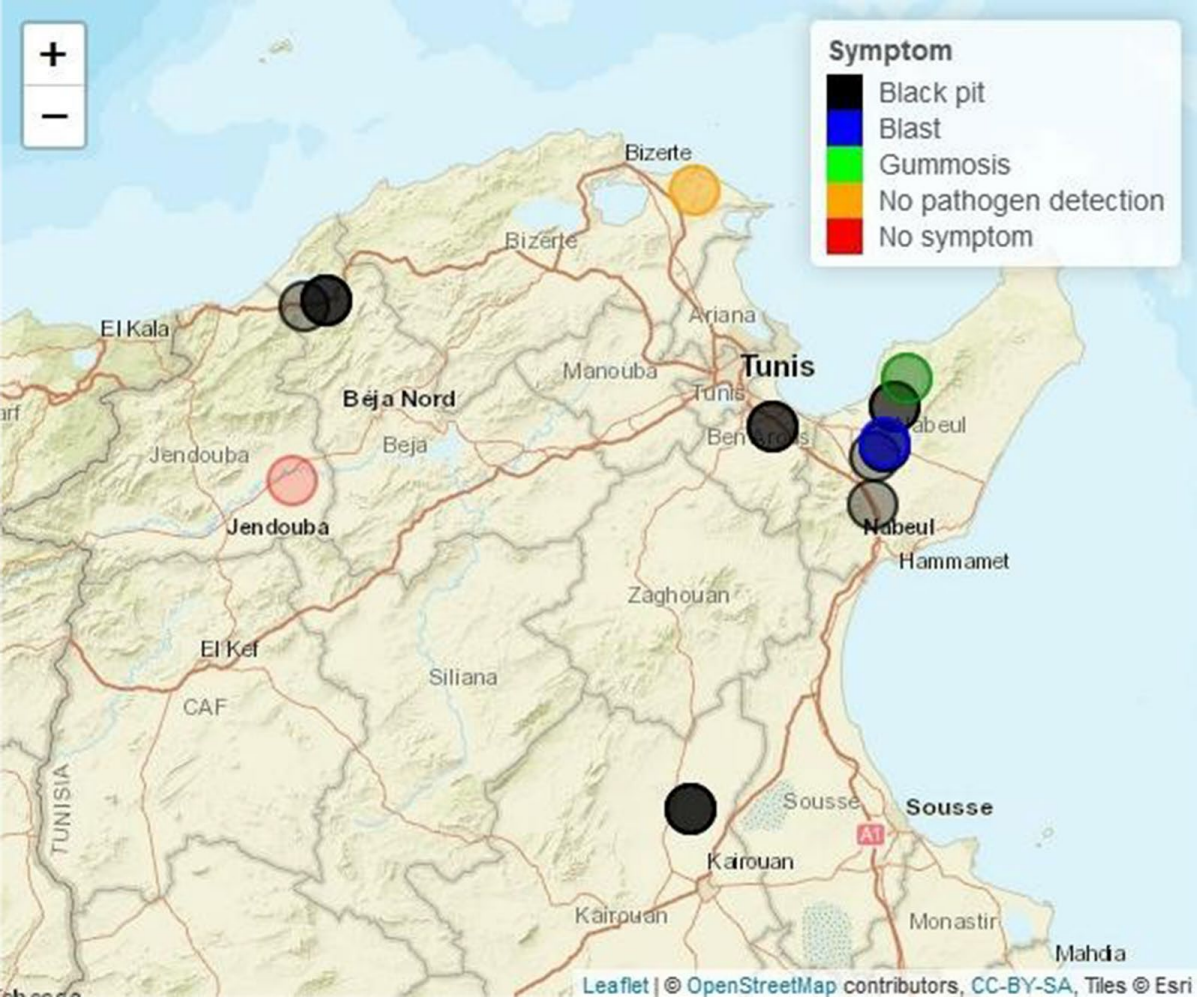
Geographical localization and distribution of symptoms in Tunisian governates sampled. No pathogen detection: Presence of symptoms but no isolation of *P. syringae*

**Fig.2:**
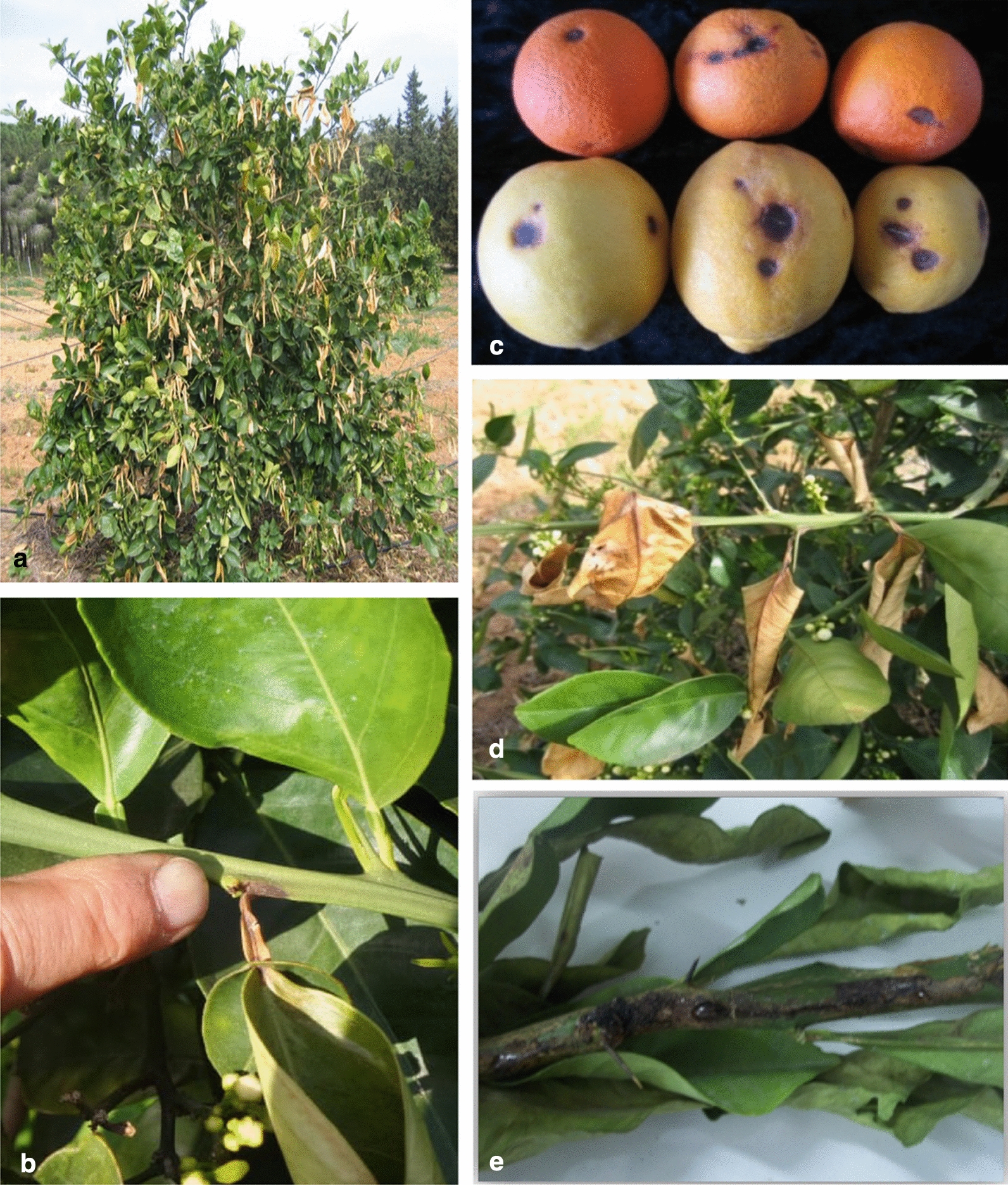
Aspect of symptoms on different plant organs and in field **a** A tree infected with blast, leaf dryness **b** Beginning of blast in petiole **c** Black pit: necrotic spots occasionally surrounded by a chlorotic halo on lemons and oranges **d** leaves dry and roll, while still firmly attached to the tree, before eventually dropping without petioles **e** Lesions and black areas on twigs

The main administrative governates (6) devoted to the production of citrus fruits in Tunisia were selected: Nabeul (Cap Bon Peninsula) located in the extreme north-east of the country, Ben Arous and Bizerte situated successively in the North and extreme north, Beja and Jendouba located in the north-west and Kairouan in the center of Tunisia (Fig. 1). A total of 37 orchards were chosen arbitrarily during winter and spring seasons of 2015, 2016 and 2017 (Additional file 1: Table S1). A survey sheet for each orchard and their characteristics was established. Depending on the incidence of the disease in the orchard, between 1 and 9 symptomatic trees were sampled, with fruit, leaf, twig or branch part of the tree being collected (Fig. 2 and Additional file 1: Table S1). The samples were taken from *C. sinensis* cvs.'Valencia Late', 'Washington Navel' and 'Maltaise', *C. limon* cvs. 'Eurêka' and 'Lunari', *C. reticulata* cvs. 'Hernandina' and ‘Cassar’ and *C. paradisi* cv.'Star Ruby’.

### Bacterial isolation

Samples were placed separately in sterile bags before being transferred to the laboratory. For each sample, small pieces of the intermediate zone between the healthy and the necrotic part were disinfected with 0.5% sodium hypochlorite and washed with sterile distilled water (SDW) macerated in phosphate buffer (K2HPO4 (BIOMATIQ): 8.75 g/l, KH2PO4 (BIOMATIQ): 6.75 g/l) and vortexed for 4 min (Monteil et al. 2011). The liquid of the crushed material was diluted to 10^–4^ and 100 µl of each sample were spread on the entire Trypticase Soy Agar (TSA, Biolife) and King B media (KB, Biolife) supplemented with cycloheximide SIGMA (200 mg/l), cephalexin SIGMA (80 mg/l) and Boric acid BIOMATIQ 1.5 g/100 ml (KBC medium). After 2 days at 28 °C, fluorescent colonies were subcultured on KB plates to ensure purity and pure strains were stored in 40% glycerol at − 80 °C and on KB at 4 °C for short-term use.

### Phenotypic characterization of strains

Colony size and morphology were determined on strains together with production of fluorescent pigments tested on King B medium (*Pseudomonas* agar F; Difco), and pyocyanin production tested on King A medium (*Pseudomonas* agar P, Biolife) (King et al. 1954). Oxidase tests were performed as previously described (Ewing et al. 1962). Strains were characterized phenotypically using API ZIM (bioMérieux) (Gruner et al. 1992) and were tested for production of syringomycin-like toxins on a minimal SRM medium (Gross 1985) that is revealed by antibiosis for *Geotrichum candidum* (Gross and Devay 1977; Morris et al. 2007). Ice nucleation activity (INA) of strains was characterized as previously described (Stopelli et al. 2014). Three replicates/strain of fresh suspensions adjusted to 10^6^ Colony-forming Unit (CFU)/ml^−1^ in phosphate buffer were used in the range of temperature from 0 to −7 °C. For all phenotypic and plant tests (see below), the pathogenic strain *P. syringae* CC94, (Morris et al. 2000), was used as a positive control and sterile distilled water as a negative control.

### Antibiotic resistance

The *Pseudomonas* isolates were evaluated against 25 antibiotics of different families using the disk diffusion method of Bauer et al. (1966). The antibiotics families used in the resistance test were: penicillin, (cephalosporin, carbapenem, aminopenicillin and aminosides), fluoroquinolone (ciprofloxacin, pefloxacin, ofloxacin), quinolone (nalidixic acid), sulfamides-trimethoprim, polypeptide, carboxypenicillin, monobactam, tetracyclines and phenicolates. Antibiotic discs were placed on nutrient agar medium (BioMérieux) previously inoculated with a pure culture of 48 h strains adjusted to 10^6^ CFU ml^−1^. After incubation at 30 °C for 24 h, inhibition zones were measured in mm, comparing them with critical values of the European Committee on Antimicrobial Susceptibility Testing (EUCAST, www.eucast.org) to evaluate whether a strain was sensitive or resistant. Multiresistance classification was performed according to Magiorakos et al. (2012). Multidrug resistance (MDR) is defined as non- susceptible to at least one agent in three or more antimicrobial categories. Extensive drug resistance (XDR) is defined as non-susceptible to at least one agent in all but two or fewer antimicrobial categories. Pandrug-resistance (PDR) is defined as non-susceptible to all agents in all microbial categories.

### Strain identification

#### Matrix-assisted laser desorption/ionisation time-of-flight mass spectrometry

Strains were identified following a chemotaxonomic approach using matrix-assisted laser desorption/ionisation time-of-flight mass spectrometry (WC MALDI-TOF MS) (Bright et al. 2002). The studied strains together with their closely related species type strains were obtained at the Scientific- Technical Services (University of Balearic Islands, Spain). Strains were cultured on LB medium at 30 °C for 24 to 48 h. After extraction, one microliter of the extract was placed onto a spot of a ground steel plate. Each sample was covered with 1 µL of matrix solution and air dried at room temperature. The measurements were carried out on an Autoflex III MALDI-TOF / TOF mass spectrometer (Bruketar Daltonics, Leipzig, Germany). Data were analyzed as previously described with an indoor database used to compare the protein profiles (Sánchez et al. 2014b).

### Phylogenetic analysis of strains

The DNA extraction, primers used, PCR amplification, and DNA sequencing conditions, as well as the sequence analysis procedures were previously described (Mulet et al. 2010). Amplified products were purified with Montage PCR filter units (Millipore) (Mulet et al. 2016). An individual tree based on the *rpoD* partial sequence alignment was generated with all studied strains (Yamamoto et al. 2000; Ghyselinck et al. 2013). A multilocus sequencing analysis (MLSA) with the concatenated three-gene- sequences 16S rRNA (1279 nt), *gyrB* (794 nt), and *rpoD* (648 nt) was also performed for those representative strains (Mulet el al. 2012). Sequences were compared with the corresponding sequences of *Pseudomonas* species type strains described up until November 2018 with 203 species type strains in our in-house database. Alignments were analyzed according to Mulet et al. (2008). The gene distances were calculated, using the Jukes-Cantor method (Jukes and Cantor 1969), and phylogenetic trees were generated by neighbour-joining using MEGA5 software (Tamura et al. 2011). Sequences obtained in this study were deposited in the EMBL database. To further identify the studied strains, a phylogenetic tree was constructed using the partial sequences of the *rpoD* gene (455 nt) using the 4 strains previously proposed (Parkinson et al. 2011; Berge et al. 2014) as reference strains to define the phylogroups PG02a, PG02b, PG02c and PG02d within the *P. syringae* species group. The 15 species type strains of the *P. syringae* group (Gomila et al. 2017) were also included in the analysis. The identity of putative *P. syringae* strains M5 and M47 from the Technical Center of Citrus orchard, Bni khalled region of Nabeul governate, and V2A2F14, 27, 45, 52 and 79 from Charfeddine orchard, Bouargoub region of Nabeul governate (Table 1), was obtained by studying their phylogenetic context. Reference strains of the *P. syringae* group of bacteria were included (see the list in Additional file 1: Fig. S1) and the analyses were based on partial sequences of the citrate synthase (*cts*) housekeeping gene as previously described (Berge et al. 2014). Primers Cts-FP (forward): 5-AGT TGA TCA TCG AGG GCG C(AT)G CC-3 and Cts-RP (reverse): 5-TGA TCG GTT TGA TCT CGC ACG G-3 (Sarkar and Guttman 2004; Morris et al. 2010) were used for DNA amplification and primer Cts-FS (fwd): 5-CCC GTC GAG CTG CCA ATW TTG CTG A-3 for sequencing. DAMBE (version 5) was used to perform sequence-alignment and Mega (version 4) to build a neighbor-joining tree. Housekeeping gene (*rpoD*) sequences available from previous studies on *P. syringae* pv. *syringae* present on citrus in Iran (FBF strains) and Montenegro (IZB strains) were compared with our data.

**Table 1:** Origin of selected strains characterized in this study

Strain^a^	Year of isolation	Governorate/region	Orchard	Cultivar	Organ	Symptom
BE12A	2017	Beja/Nefza	M. Guennouni^d^	*C*^*e*^*. sinensis* 'Valencia Late'	Fruit	Black pit
BE1	2017	Beja/Nefza	M. Guennouni	*C. sinensis* 'Valencia Late'	Fruit	Black pit
BE3	2017	Beja/Nefza	M. Guennouni	*C. sinensis* 'Valencia Late'	Fruit	Black pit
Iy3DA	2017	Ben Arous/Naasen	Ayari	*C. limon* 'Eurêka'	Fruit	Black pit
Iy3EA	2017	Ben Arous/Naasen	Ayari	*C. limon* 'Eurêka'	Fruit	Black pit
Iy3GB	2017	Ben Arous/Naasen	Ayari	*C. limon* 'Eurêka'	Fruit	Black pit
Iy5HA	2017	Ben Arous/Naasen	Ayari	*C. limon* ' Eurêka '	Fruit	Black pit
IyGA	2017	Ben Arous/Naasen	Ayari	*C. sinensis* 'Maltaise'	Fruit	Black pit
IyGC	2017	Ben Arous/Naasen	Ayari	*C. sinensis* 'Maltaise'	Fruit	Black pit
KB49	2017	Kairouan/Sbikha	Chaabani 1	*C. sinensis* 'Valencia Late'	Fruit	Black pit
KC19	2017	Kairouan/Sbikha	Chaabani 2	*C. sinensis* 'Valencia Late'	Fruit	Black pit
KC29B2	2017	Kairouan/Sbikha	Chaabani 2	*C. sinensis* 'Valencia Late'	Fruit	Black pit
KC46	2017	Kairouan/Sbikha	Chaabani 2	*C. sinensis* 'Valencia Late'	Fruit	Black pit
KC54	2017	Kairouan/Sbikha	Chaabani 2	*C. sinensis* 'Valencia Late'	Fruit	Black pit
KC55	2017	Kairouan/Sbikha	Chaabani 2	*C. sinensis* 'Valencia Late'	Fruit	Black pit
KC82	2017	Kairouan/Sbikha	Chaabani 2	*C. sinensis* 'Valencia Late'	Fruit	Black pit
TRR12	2015	Nabeul/Bouargoub	The Gardens	*C. limon’*Lunari’	Fruit	Black pit
TRR15	2015	Nabeul/Bouargoub	The Gardens	*C. limon’*Lunari’	Fruit	Black pit
TRR9	2015	Nabeul/Bouargoub	The Gardens	*C. limon’*Lunari’	Fruit	Black pit
MTR3B	2015	Nabeul/Tekelsa	SOTAM^c^	*C. reticulata*’Hernandina’	Branch	Gummosis
E10AA	2017	Nabeul/Bnikhalled	Chakib	*C. limon* 'Eurêka'	Fruit	Black pit
E10CA	2017	Nabeul/Bnikhalled	Chakib	*C. limon* 'Eurêka'	Fruit	Black pit
E10CB2	2017	Nabeul/Bnikhalled	Chakib	*C. limon* 'Eurêka'	Fruit	Black pit
E11	2017	Nabeul/Bnikhalled	Chakib	*C. limon* 'Eurêka'	Fruit	Black pit
E12A	2017	Nabeul/Bnikhalled	Chakib	*C. limon* 'Eurêka'	Fruit	Black pit
E91	2016	Nabeul/Bnikhalled	Dhaouadi	*C. limon* 'Eurêka'	Twig	Blast
E93A	2016	Nabeul/Bnikhalled	Dhaouadi	*C. limon* 'Eurêka'	Twig	Blast
E9A	2016	Nabeul/Bnikhalled	Dhaouadi	*C. limon* 'Eurêka'	Twig	Blast
EL1A	2016	Nabeul/Bnikhalled	TCC^b^	*C. limon* 'Eurêka'	Fruit	Black pit
EL2	2016	Nabeul/Bnikhalled	TCC	*C. limon* 'Eurêka'	Fruit	Black pit
M5*	2016	Nabeul/Bnikhalled	TCC	*C. reticulata ‘*Cassar’	Fruit	Black pit
M29*	2016	Nabeul/Bnikhalled	TCC	*C. reticulata* ‘Cassar’	Fruit	Black pit
M30*	2016	Nabeul/Bnikhalled	TCC	*C. reticulata ‘*Cassar’	Fruit	Black pit
M32*	2016	Nabeul/Bnikhalled	TCC	*C. reticulata* ‘Cassar’	Fruit	Black pit
M47*	2016	Nabeul/Bnikhalled	TCC	*C. reticulata* ‘Cassar’	Fruit	Black pit
V2A2F14*	2016	Nabeul/Bouargoub	Charfeddine	*C. sinensis* 'Maltaise'	Fruit	Black pit
V2A2F15*	2016	Nabeul/Bouargoub	Charfeddine	*C. sinensis* 'Maltaise'	Fruit	Black pit
V2A2F27*	2016	Nabeul/Bouargoub	Charfeddine	*C. sinensis* 'Maltaise'	Fruit	Black pit
V2A2F45*	2016	Nabeul/Bouargoub	Charfeddine	*C. sinensis* 'Maltaise'	Fruit	Black pit
V2A2F52*	2016	Nabeul/Bouargoub	Charfeddine	*C. sinensis* 'Maltaise'	Fruit	Black pit
V2A2F79*	2016	Nabeul/Bouargoub	Charfeddine	*C. sinensis* 'Maltaise'	Fruit	Black pit

^a^ Strainwith * were lost during thestudy and were no longer available to do all tests. All strains were fluorescent on KB medium and negativefor oxidase

^b^ Technical Center of Citrus

^c^ Tunisian Society of Modern Agriculture

^d^ M. Guennouni:MiledGuennouni

^e^ *Citrus*

### Nucleotide sequence numbers

The GenBank/EMBL/DDBJ accession numbers for the nucleotide sequences reported in this study are as follows: LR214467-LR214509 for the *rpoD* gene, LR214451-LR214466 for the 16S rRNA and LR214510-LR214525 for the *gyrB* gene.

### Tests on plants

Pathogenicity tests were performed on lemon fruits and detached leaves of *C. limon* cv. 'Eurêka'. Fruits and leaves were dipped in a solution of sodium hypochlorite (1% active hypochlorite) for 5 min, rinsed three times in sterile distilled water and gently dried with filter paper. The pathogenic strain *P. syringae* pv. *syringae* KB49 (a member of phylogroup PG02b) isolated from a citrus orchard in Tunisia with black pit symptoms was used as a positive control. Strains were grown on Luria Broth medium at 28 °C for 48 h. One or two colonies were suspended in sterile distilled water to an absorbance at 580 nm between 0.06 and 0.12, which corresponds to approximately 10^8^ CFU/ml. The suspensions (10 μl) were inoculated either by injection with a tuberculin needle at the fruit wall or by spraying on the surface of fruits. Negative controls were treated similarly with sterile distilled water (Gilbert et al. 2010). Three fruits were inoculated for each strain as well as for the controls. After 5 days at 20 ℃, necroses were measured for both methods of inoculation (Iacobellis et al. 1994). For leaves, 10 µl of the bacterial suspension were injected into the central veins of the abaxial side and inoculated leaves were incubated. The experiment consisted of three replicates per strain. To verify Koch Postulate, bacteria were reisolated on KB medium and BOX elements were used for DNA fingerprinting (Marques et al. 2008; Koeuth et al. 1995).

*Cucumis melo* var. *cantalupensis* Naud. cv. Védrantais seedlings were used as an indicator plant to estimate the level of aggressiveness (Morris et al. 2000). These parameters were assessed after infiltrating twelve seedlings at the junction of the cotyledons with 10 µl of an aqueous bacterial suspension (10^8^ CFU ml^−1^) prepared from 48 h bacterial cultures (Morris et al. 2008). Seedlings were incubated for seven days with photoperiod of 16 h of light at 21 °C during the day and 18 °C during the dark period. Symptoms on seedlings were scored as follows: 0 (no symptoms), 1 (one cotyledon with necrosis or completed wilted), 2 (necrosis on both cotyledons), 3 (both cotyledons wilted and stem symptoms) and 4 (death of the entire plantlet). Pathogenicity was recorded positive when the frequency of seedlings with symptoms (F) was  > 50% and aggressiveness was calculated as the mean score of symptoms (µ) (Berge et al. 2014). Sterile distilled water was used as a negative control and *P. syringae* CC94 as a positive control.

The capacity of strains to induce a hypersensitive response (HR) was determined in tobacco by infiltrating fully developed leaves of plants of *Nicotiania tobacum* L. cv. Samsun at the 10-leaf stage (bacterial suspensions of 48 h cultures at approximately 10^8^ CFU ml^–1^) (Leliott et al. 1966).

## Results

Thirty-seven orchards of citrus implanted in fourteen regions of six different governates located in northern Tunisia were surveyed for black pit and blast symptoms during three seasons (2015–2017). Tentative isolations of pathogenic *Pseudomonas* sp. were performed from 279 samples collected on symptomatic trees (Fig. 2 and Additional file 1: Table S1). The percentage of symptomatic trees in these orchards was less than 5% indicating a low incidence of disease. However in two orchards, this percentage was higher (19 and 22.5%) showing these diseases could be very serious. It is important to perform isolation of pathogenic bacteria from orchards that will help when present, to avoid dissemination. The prevalence of the disease varies by governate (Fig. 1). In Jendouba governate, no symptoms were observed in two orchards from the Bouselem region surveyed in 2017. In the three orchards from the Ras Jbel region of Bizerte governate symptoms were observed, especially in Ras Jbel 1 orchard in 2015 (22.5% of symptomatic trees) but pathogenic *Pseudomonas* sp. were not isolated from the 41 samples collected (Additional file 1: Table S1).

In Kairouan governate, found free from disease in a 2012–15 survey (Abdellatif et al. 2017), symptoms in the two orchards from Sbikha region were observed in 2017, and pathogenic *Pseudomonas* sp. were isolated from both of them. In Ben Arous and Beja governates, symptoms were present in all seven orchards studied however, pathogenic *Pseudomonas* sp. were isolated only from one orchard per governate: Ayari orchard, Naasen region of Ben Arous, and Miled Guennouni orchard, Nefza region of Beja, both surveyed in 2017. Finally, in Nabeul governate where the survey was the largest, symptoms were present in 20 orchards among the 24 visited in 6 regions during the three years of the study. In this governate, pathogenic *Pseudomonas* sp. was isolated from 6 symptomatic orchards located in BniKhalled (3), Tekelsa (1) and Bouargoub (2) regions (Additional file 1: Table S1).

All orchards free of symptoms were conduced following conventional methods with irrigation either by surface or drip technique. Pathogenic *Pseudomonas* sp. strains were isolated in 44% of conventional and 25% of organic symptomatic orchards surveyed (Additional file 1: Table S1). Our extensive study confirms the presence of symptomatic trees in many Tunisian citrus orchards and describe for the first time in 2017, symptoms in orchards of the Kairouan region (Fig. 1). Moreover this work shows that the disease affects many different citrus cultivars grown in Tunisia. A total of 820 strains were isolated from 81 symptomatic trees and based on colony morphology, 54 putative *P. syringae* strains were selected to be characterized (Additional file 1: Table S1).

### Strain identification

Among the 54 putative *P. syringae*, 11 strains isolated in 2016, fluorescent on KB medium and tested negative for cytochrome c oxidase activity, were firstly identified using partial housekeeping *cts* gene sequencing following Berge et al. (2014). Five strains from Technical Center of Citrus sharing the same BOX-PCR profile and 6 strains from Charfeddine orchards (Nabeul) were affiliated to *P. syringae* (phylogroup PG02b) and *P. cerasi* (phylogroupPG02a) respectively (Tables 1 and 2; Additional file 1: Fig. S1). These two species belong to the *P. syringae* group of bacteria (Gomila et al. 2017).

**Table 2:** Identification and classification of strains based on WC-MALDI-TOF MS and on phylogenetic analysis of housekeeping genes sequences

Strain^a^	WC-MALDI-TOF-MS^b^		Sequences identity				Final identi-fication	*P. s.* phylo-group^f^	Box-PCR profile^g^
	Best match with	Score value^d^	*cts*or*rpoD* gene sequence		Concanated gene sequence^e^				
			Closest type strain	%	Closest type strain	%			
BE12A	*P. congelans*	2.09	*P. congelans(rpoD)*	98.0	*P. congelans*	99.1	*P. congelans*	PG02c	NA
BE1	*P. syringae*	2.06	*P. syringae (rpoD)*	99.7	*P. syringae*	99.6	*P. syringae*	PG02b	P1
BE3	*P. syringae*	2.01	*P. syringae (rpoD)*	99.8	*P. syringae*	99.6	*P. syringae*	PG02b	P2
Iy3DA	*P. syringae*	1.75	*P. syringae (rpoD)*	99.5	NA		*P. syringae*	PG02b	NA
Iy3EA	*P. syringae*	1.98	*P. syringae (rpoD)*	99.7	NA		*P. syringae*	PG02b	NA
Iy3GB	*P. syringae*	1.83	*P. syringae (rpoD)*	99.7	NA		*P. syringae*	PG02b	NA
Iy5HA	*P. syringae*	2.33	*P. syringae (rpoD)*	99.7	*P. syringae*	99.2	*P. syringae*	PG02b	NA
IyGA	*P. syringae*	1.93	*P. syringae (rpoD)*	99.5	NA		*P. syringae*	PG02b	NA
IyGC	*P. syringae*	2.36	*P. syringae (rpoD)*	99.7	NA		*P. syringae*	PG02b	P2
KB49	*P. cannabina/P. syringae*	1.99/1.87	*P. syringae (rpoD)*	99.7	NA		*P. syringae*	PG02b	P2
KC19	*P. syringae*	2.11	*P. syringae (rpoD)*	99.7	NA		*P. syringae*	PG02b	NA
KC29B2	*P. syringae*	1.84	*P. syringae (rpoD)*	99.5	NA		*P. syringae*	PG02b	P2
KC46	*P. syringae*	2.01	*P. syringae (rpoD)*	99.5	NA		*P. syringae*	PG02b	NA
KC54	*P. syringae*	1.94	*P. syringae (rpoD)*	99.7	NA		*P. syringae*	PG02b	P5
KC55	*P. syringae*	2.13	*P. syringae (rpoD)*	99.7	NA		*P. syringae*	PG02b	NA
KC82	*P. syringae*	1.99	*P. syringae (rpoD)*	99.7	NA		*P. syringae*	PG02b	NA
TRR12	*P. syringae*	2.06	*P. syringae (rpoD)*	99.8	*P. syringae*	99.6	*P. syringae*	PG02b	P2
TRR15	*P. syringae*	2.15	*P. syringae (rpoD)*	99.9	NA		*P. syringae*	PG02b	P2
TRR9	*P. syringae*	2.02	*P. syringae (rpoD)*	99.8	*P. syringae*	99.6	*P. syringae*	PG02b	P2
MTR3B	*P. congelans*	2.12	*P. congelans(rpoD)*	98.2	NA		*P. congelans*	PG02c	P7
E10AA	*P. syringae*	1.92	*P. syringae (rpoD)*	99.7	NA		*P. syringae*	PG02b	P4
E10CA	*P. syringae*	2.42	*P. syringae (rpoD)*	99.8	NA		*P. syringae*	PG02b	P2
E10CB2	*P. syringae*	2.45	*P. syringae (rpoD)*	99.7	*P. syringae*	99.2	*P. syringae*	PG02b	P2
E11	*P. syringae*	2.19	*P. syringae (rpoD)*	99.5	*P. syringae*	99.5	*P. syringae*	PG02b	NA
E12A	*P. syringae*	2.17	*P. syringae (rpoD)*	99.7	*P. syringae*	99.6	*P. syringae*	PG02b	NA
E91	*P. syringae*	2.21	*P. syringae*(*rpoD)*	99.5	NA		*P. syringae*	PG02b	P3
E93A	*P. syringae*	2.46	*P. syringae*(*rpoD)*	97.9	NA		*P. syringae*	PG02b	P6
E9A	*P. syringae*	2.43	*P. syringae*(*rpoD)*	99.7	NA		*P. syringae*	PG02b	NA
EL1A	*P. syringae*	2.11	*P. syringae*(*rpoD)*	99.4	*P. syringae*	99.1	*P. syringae*	PG02b	NA
EL2	*P. syringae*	1.97	*P. syringae*(*rpoD)*	99.5	*P. syringae*	99.3	*P. syringae*	PG02b	NA
M5*	NA^c^	NA	*P. syringae*(*cts*)		NA		*P. syringae*	PG02b	P8
M29*	NA	NA	NA		NA		*P. syringae*	PG02b	P8
M30*	NA	NA	NA		NA		*P. syringae*	PG02b	P8
M32*	NA	NA	NA		NA		*P. syringae*	PG02b	P8
M47*	NA	NA	*P. syringae*(*cts*)		NA		*P. syringae*	PG02b	P8
V2A2F14*	NA	NA	*P. cerasi*(*cts*)		NA	NA	*P. cerasi*	PG02a	NA
V2A2F15*	NA	NA	*P. cerasi*(*cts*)		NA		*P. cerasi*	PG02a	NA
V2A2F27*	NA	NA	*P. cerasi*(*cts*)		NA		*P. cerasi*	PG02a	NA
V2A2F45*	NA	NA	*P. cerasi*(*cts*)		NA		*P. cerasi*	PG02a	NA
V2A2F52*	NA	NA	*P. cerasi*(*cts*)		NA		*P. cerasi*	PG02a	NA
V2A2F79*	NA	NA	*P. cerasi*(*cts*)		NA	NA	*P. cerasi*	PG02a	NA

^a^Strainwith * werelostduringthestudy and were no longeravailable to do alltests

^b^ Biotyper-indoor DB

^c^ NA, Not Analysed

^d^ Score value between 2.0 and 2.3 indicates a secure identification at the genus level, but not at the species level; a value equal orhigher than 2.3 indicates a secure species identification; values lower than 2.0 indicates probably identification at the genus level

^e^ Concatenated gene sequences**:** partialsequences of *rrs* (16S rRNA*), gyrB*and *rpoD*genes wereconcatenatedbeforephylogeneticanalysis

^f^ *P. s.* phylogroup, *P. syringae*phylogroup were assigned following Berge et al. (2014)

^g^ Box-PCR profile of strains were visually compared. Strains sharing the same Box-PCR profile were considered very close and are supposed to belong to the same species and phylogroup (see Additional file 1: Figure S1 to see P1 to P7)

These 43 strains were tested Gram negative and they were able to grow and fluoresce on KB and KBC media. The oxidase test was positive for 13 strains and negative for 30 strains. *P. syringae* do not have cytochrome c oxidase and the work was focused on the 30 strains negative for oxidase (Table 1), the 15 remaining strains being kept in the study to look at their identification. WC MALDI-TOF MS protein profiles were obtained for all 43 strains which were identified with good confident values (≥ 2) in the Biotyper database (Table 2 and Additional file 1: Table S2).

Their chemotaxonomic analysis classified all of them but one, in the fluorescent *Pseudomonas* lineage of Mulet et al. (2010) classification. The 13 oxidase positive strains were identified to *P. lactis* (2 strains from the same tree), a species of the *P. fluorescens* subgroup of that lineage (Additional file 1: Table S2), and to *P. moraviensis* (10 strains from 4 different orchards), a species of the *P. koreensis* subgroup previously isolated and tested pathogenic on citrus in Iran (Beiki et al. 2016). One strain was affiliated to the *P. orizyhabitans* species, a *Pseudomonas* lineage distant of the other *Pseudomonas*. These species were non-pathogenic on cantaloupe (one strain per species tested) and citrus (one *P. moraviensis* strain tested) (Additional file 1: Table S2). As expected, the 30 oxidase negative strains were affiliated to *P. syringae* (PG02b) (28 strains) and *P. congelans* (PG02c) (2 strains) (Tables 2 and Additional file 1: S1). These two species are included in the *P. syringae* group of bacteria (Gomila et al. 2017).

**Fig.3:**
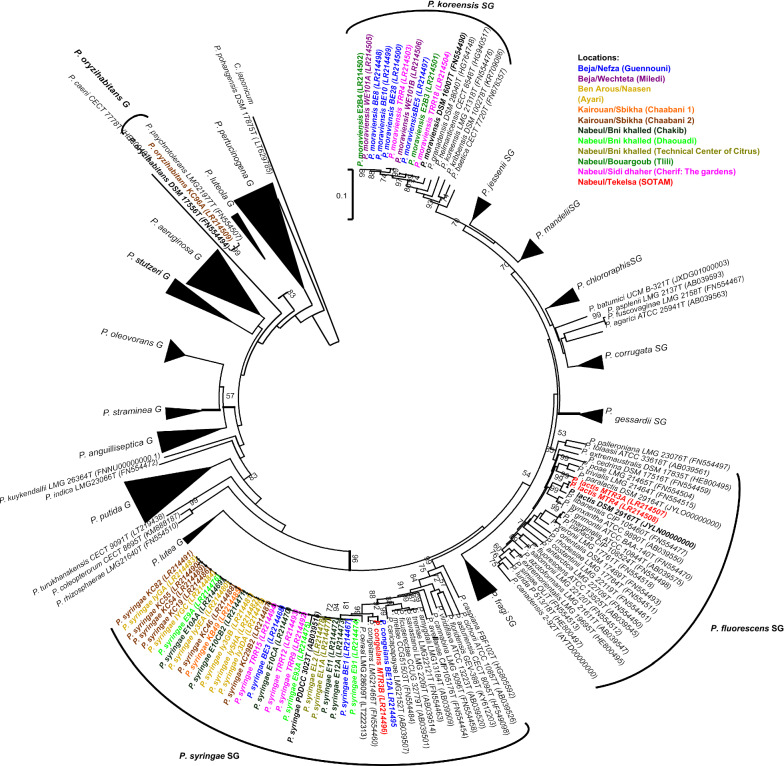
Phylogenetic tree of isolated strains and 203 *Pseudomonas* species type strains, based on the partial *rpoD* sequence analysis. Distance matrices were calculated by the Jukes-Cantor method (Jukes and Cantor 1969). Dendrograms were generated by the neighbour-joining method. The *Cellvibrio japonicus* Ueda107 *rpoD* sequence was used as the outgroup. The bar indicates sequence divergence. Percentage bootstrap values of more than 50% (from 1000 replicates) are indicated at the nodes. GenBank accession numbers are given in parentheses. Colors indicate the sampling locations

**Fig.4:**
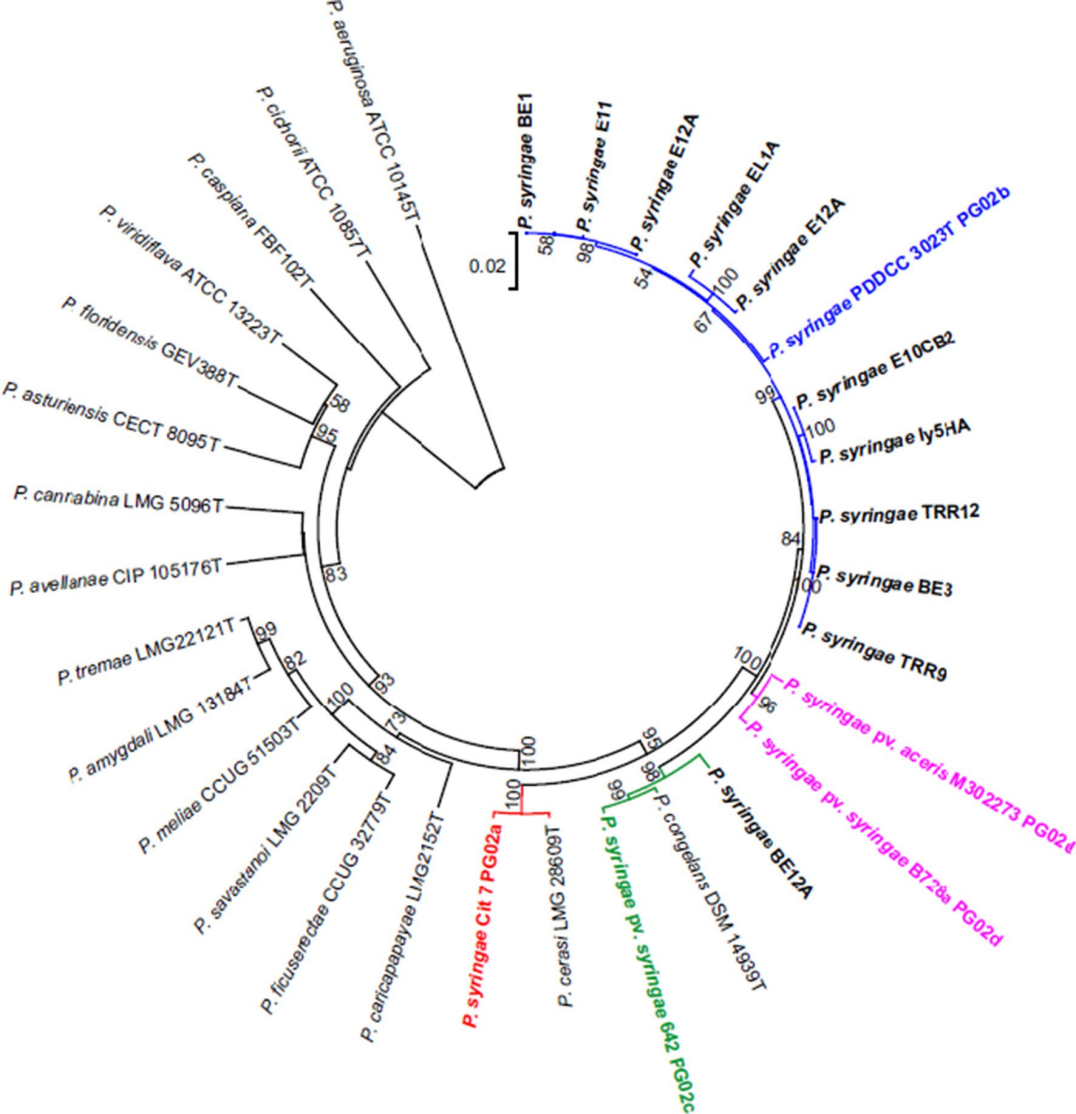
Phylogenetic tree of representative strains of the *P. syringae* group isolated in this study from citrus plants with symptoms. Concatenated 16S rRNA, *gyrB* and *rpoD* gene partial sequences were used and reference strains of the *P. syringae* phylogenetic group were included in the analysis. Distance matrices were calculated by the Jukes-Cantor method (Jukes and Cantor 1969). The tree was generated by the neighbour-joining method. *P. aeruginosa* ATCC 10145^ T^ was used as the outgroup. The bar indicates sequence divergence. Percentage bootstrap values of more than 50% (from 1000 replicates) are indicated at the nodes

Phylogenetic analyses were performed to confirm identification and to know how closely related the strains were. Analysis of the partial *rpoD* gene sequences of all 43 strains was consistent with the chemotaxonomic analysis results. A phylogenetic tree of these sequences including 203 *Pseudomonas* type strains sequences from an in-house database was constructed. It confirmed the MALDI-TOF MS identification and illustrated the strain positions in the different *Pseudomonas* lineages (Fig. 3). The 30 strains affiliated with the *P. syringae* group showed high percentages of identity with *P. syringae* (PG02b) and *P. congelans* (PG02c) type strains as expected (Table 2). Some representative strains of the *P. syringae* group were selected to refine the *rpoD* analysis by doing a multilocus sequence analysis (MLSA) of concatenated sequences of the housekeeping genes *rpoD*, *gyrB* and 16S rRNA as depicted in Table 2. The topology of the tree obtained (Fig. 4) was similar to that of the *rpoD* gene and identification of strains was definitely confirmed (Table 2). PG02b strains exhibited different haplotypes of concatenated sequences (Fig. 4) showing they were not clonal. This was confirmed by the various BOX- PCR profiles obtained for some strains (Additional file 1: Fig. S2 and Table 2).

Some enzymatic activities were tested on the 30 strains from the *P. syringae* group and were quite homogeneous whether for *P. syringae* (PG02b) or *P. congelans* (PG02c) strains (Additional file 1: Table S3). By contrast, antibiotic resistance patterns were more variable. Among the 14 strains tested, 11 were resistant at least to one antibiotic family (Additional file 1: Table S4). Nine and 6 strains were resistant to aztreonam (ATM) and gentamicin (GM) respectively, and 4 strains were resistant to both of them. Strains of *P. congelans* (PG02c) showed larger patterns of resistance than those of *P. syringae* strains. Moreover, *P. congelans* MTR3B appeared multi drug resistant (MDR3) to ATM, GM and imipenem (IMP) (Additional file 1: Table S4). Our study led to the isolation of diverse strains of the *P. syringae* group of bacteria from symptomatic citrus trees. In order to know which strains could be involved in the citrus disease in Tunisia, their pathogenicity was tested.

### Pathogenicity tests

**Fig.5:**
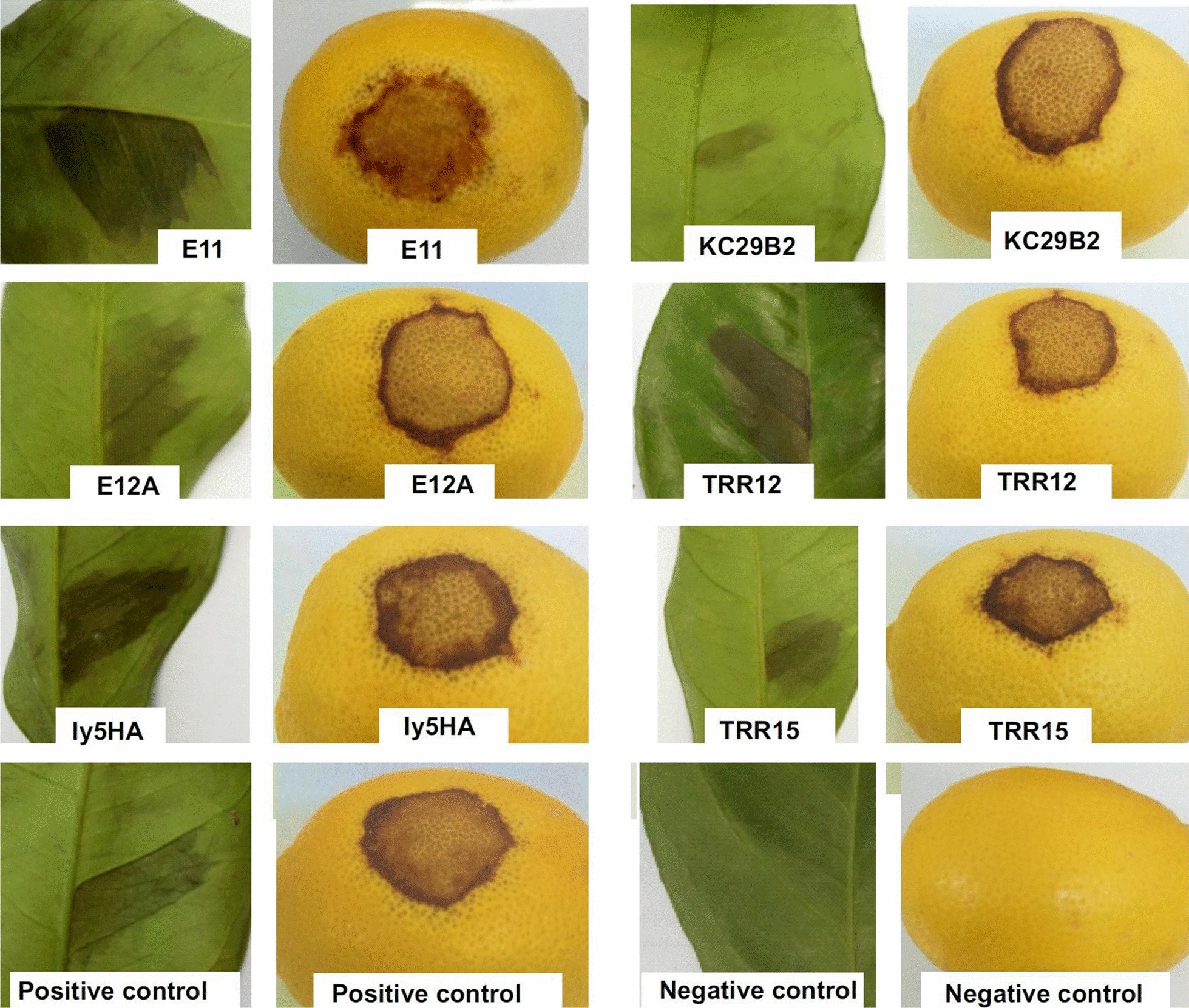
Detail of necrotic spots on leaves and fruits of *Citrus limon* cv. ‘Eurêka’ induced by artificial inoculation with *P. syringae* pv. *syringae*. Black pit on fruits 5 days after inoculation and blast on leaves, 24 h later as compared with negative control (treated with sterile distilled water)

A selection of 37 representative strains (30 *P. syringae* (PG02b), 6 *P. cerasi* (PG02a) and 1 *P. congelans* (PG02c)) was included in these tests (Table 3). All the *P. syringae* (PG02b) strains injected in lemon fruits produced clear brown spots on the surface with diameters from 1.3 to 4.1 cm (Fig. 5 and Table 3). In the tests without injection, they produced necrosis lesion areas on fruits or leaves but were smaller in diameter (0.2 to 2.5 cm) and 3 strains were negative (Table 3).

**Table 3:** Strain characteristics potentially involved in pathogenicity on plants

Strains^a^	Taxonomic position	Pathogenicity on lemon^c^			Pathogenicity on cantaloupe^e^			HR^f^	Toxin produc-tion^g^		INA^h^	
		Necrosis diameter after injection in fruit(cm)	Necrosis diameter after spraying fruit (cm)	Necrosis surface after infiltration of leaves (cm^2^)	Mean symptom rate	F. of plants with rate > 1	Agressivness	HR ontobacco	Inhibition zone (cm)	Syringomycinproduction	Freezing Test (°C)	Ice nucleationactivity
BE12A	*P. congelans*(PG02c)	0.1 ± 0.1	0.0	0	3.3	1.0	+	±	0.6	+	− 1.5	+
BE1	*P. syringae* (PG02b)	1.7 ± 0.4	0.8 ± 0.5	0.9	3.6	1.0	+	+	1.1	+	− 1.5	+
BE3	*P. syringae* (PG02b)	3.2 ± 0.4	1.0 ± 0.3	1.2	3.5	1.0	+	+	1.1	+	− 2.1	+
ly3DA	*P. syringae* (PG02b)	1.9 ± 0.2	0.2 ± 0.1	1.0	3.6	1.0	+	+	1.3	+	− 2.6	+
ly3EA	*P. syringae* (PG02b)	3.9 ± 0.4	1.2 ± 0.2	1.1	2.3	1.0	+	+	1.2	+	− 1.5	+
ly3GB	*P. syringae* (PG02b)	2.7 ± 0.1	0.8 ± 0.1	1.3	2.5	0.9	+	+	1.3	+	− 2.6	+
ly5HA	*P. syringae* (PG02b)	2.1 ± 1.0	0.7 ± 0.2	1.0	3.8	1.0	+	+	1.2	+	− 1.5	+
lyGA	*P. syringae* (PG02b)	2.6 ± 0.1	0.8 ± 0.2	1.0	2.8	1.0	+	+	1.2	+	− 1.4	+
lyGC	*P. syringae* (PG02b)	3.0 ± 0.1	1.2 ± 0	0.9	0.0	0.0	−	+	1.2	+	− 1.5	+
KB49	*P. syringae* (PG02b)	2.3 ± 0.4	1.4 ± 0.3	1.0	3.5	1.0	+	+	1.1	+	− 2.5	+
KC19	*P. syringae*(PG02b)	2.8 ± 0.4	1.6 ± 0.2	1.0	3.3	1.0	+	+	1.2	+	− 1.9	+
KC29B2	*P. syringae*(PG02b)	3.3 ± 0.4	0.9 ± 0.1	1.1	3.1	1.0	+	+	1.3	+	− 1.7	+
KC46	*P. syringae* (PG02b)	3.3 ± 0.3	0.0	0.8	3.7	1.0	+	+	1.2	+	− 2.2	+
KC54	*P. syringae* (PG02b)	3.4 ± 0.2	1.1 ± 0.4	1.1	3.8	1.0	+	+	1.1	+	− 1.2	+
KC55	*P. syringae* (PG02b)	3.5 ± 0.8	1.6 ± 0.2	0.9	2.8	0.8	+	+	1.3	+	− 1.4	+
KC82	*P. syringae* (PG02b)	3.8 ± 0.4	0.5 ± 01	1.0	3.7	1.0	+	+	1.6	+	− 1.6	+
TRR12	*P. syringae* (PG02b)	2.5 ± 0.4	0.0	1.0	2.7	1.0	+	+	1.3	+	− 1.4	+
TRR15	*P. syringae* (PG02b)	2.5 ± 0.3	0.7 ± 0.1	1.2	2.8	0.9	+	+	1.3	+	− 2.4	+
TRR9	*P. syringae* (PG02b)	2.7 ± 0.2	0.3 ± 0.4	0.9	2.8	1.0	+	+	1.4	+	− 1.6	+
E10AA	*P. syringae* (PG02b)	1.8 ± 0.3	1.2 ± 0.1	1.5	3.2	1.0	+	+	1	+	− 1.3	+
E10CA	*P. syringae* (PG02b)	2.8 ± 0.2	0.1 ± 0.1	0.8	3.0	1.0	+	+	0.7	+	− 2.0	+
E10CB2	*P. syringae*(PG02b)	1.8 ± 0.2	0.6 ± 0.3	1.2	3.5	1.0	+	+	1.2	+	− 1.8	+
E11	*P. syringae* (PG02b)	2.7 ± 0.6	1.4 ± 0.1	1.1	2.3	0.6	+	+	1.2	+	− 2.1	+
E12A	*P. syringae* (PG02b)	2.6 ± 0.1	2.5 ± 0.3	1.2	3.2	1.0	+	+	1.1	+	− 1.6	+
E91	*P. syringae* (PG02b)	3.0 ± 0.4	1.9 ± 0.4	1.0	3.8	1.0	+	+	1.1	+	− 1.8	+
E93A	*P. syringae* (PG02b)	2.0 ± 0.4	0.5 ± 0.2	1.1	3.0	0.8	+	+	0.9	+	− 2.3	+
E9A	*P. syringae* (PG02b)	3.0 ± 0.3	2.2 ± 0.3	0.7	3.7	1.0	+	+	1.5	+	− 1.2	+
EL1A	*P. syringae* (PG02b)	2.2 ± 0.3	0.0	1.0	2.9	0.9	+	+	0.7	+	−1.2	+
EL2	*P. syringae* (PG02b)	4.1 ± 0.2	0.8 ± 0.3	0.8	2.7	0.9	+	+	0.6	+	− 0.9	+
M5*	*P. syringae* (PG02b)	1.5 ± 0.1	ND	ND	ND	ND	ND	ND	ND	ND	ND	ND
M47*	*P. syringae* (PG02b)	1.6 ± 0.4	ND	ND	ND	ND	ND	ND	ND	ND	ND	ND
V2A2F14*	*P. cerasi*(PG02a)	1.1 ± 0.3	ND	ND	2.5	0.9	+	−	0.0	−	ND	ND
V2A2F15*	*P. cerasi*(PG02a)	ND	ND	ND	ND	ND	ND	−	0.0	−	ND	ND
V2A2F27*	*P. cerasi*(PG02a)	1.2 ± 0.1	ND	ND	2.3	0.9	+	−	0.2	±	ND	ND
V2A2F45*	*P. cerasi*(PG02a)	1.3 ± 0.4	ND	ND	0.0	0	−	−	1.5	+	ND	ND
V2A2F52*	*P. cerasi*(PG02a)	ND	ND	ND	0.1	0.1	−	−	0.0	−	ND	ND
V2A2F79*	*P. cerasi*(PG02a)	ND	ND	ND	0.0	0.0	−	−	0.0	−	ND	ND
CC94^b^	*P. syringae*(PG02d)	ND	ND	ND	2.7	1.0	+	+	1.2	+	− 3.2	+
Water^b^	−	0	0	0	0.0	0	−	−	0	−	> 10	−

^a^ Strainwith * were lost during the study and were no longer available to do all tests

^b^ CC94: positive control. Water: negative control

^c^ Necrotic diameters on fruits were measured in cm, lesion areas on leaves were expressed in cm^2^ (average of three replicates ± standard deviation)

^d^ ND, not determined

^e^ Symptom intensity on cantaloupe was rated on 12 seedlingsfrom 0 (no symptom) to 4 (dead plantlet) seven days after inoculation with 10^6^ cells. F. of plants with rate > 1: Frequency of plants with rate > 1

^f^ HR, Hypersensitive Response of tobacco after infiltration of a leaf with 10^6^ bacteria. The reading was done 48 h after infiltration^g^ Production of syringomycine-like toxins was tested on agar medium using*Geotrichumalbicans*as a test organism

^h^ INA, Ice Nucleation Activity was tested with bacterial suspension at 10^6^cells/ml

Agressivity of strains was quantified on cantaloupe plantlets. All 30 *P. syringae* (PG02b) strains but one (lyGC) exhibited quite high agressivity compared to the control strain CC94 known to be aggressive on this plant (Morris et al. 2000). All *P. syringae* (PG02b) strains triggered hypersensitive response on tobacco, and produced syringomycine-like toxins. These traits could be involved in the pathogenicity on citrus. Moreover they tested positive for ice nucleative activity at relatively warm temperatures (− 2.6 to–1.2 °C) which could increase the pathogeny, in the case of late frost. By contrast, the strain *P. congelans* (PG02c) BE12A produced negligible necrosis in injected lemon fruit (0, 9 cm) and showed no effect when sprayed on the lemon fruit surface or infiltrated in lemon leaves (Table 3). It could be considered as non-pathogenic on citrus. Test HR on tobacco was not clearly positive and it produced a very small amount of syringomycin-like toxins. However, it was aggressive on cantaloupe and active for ice nucleation at − 1.5 °C.

The 3 *P. cerasi* (PG02a) strains tested on citrus fruit after injection produced clear necroses but smaller than those produced by *P. syringae* (PG02b) (Table 3). Two of these 3 strains were aggressive on cantaloupe, the last and two other strains being non-pathogenic on this plant. All the six strains of *P. cerasi* (PG02a) tested on tobacco were negative and only one of them clearly produced syringomycin-like toxin. This last strain was not aggressive on cantaloupe. The pathogenicity of this group of bacteria was variable and strain dependent.

### Comparison of isolated strains with other *P. syringae* strains isolated on symptomatic citrus from other locations

The phylogenetic position of our *P. syringae* strains (30 strains) was compared with those isolated on citrus in Montenegro (12 IZB strains) and Iran (73 FBF strains) using *rpoD* gene sequences (Additional file 1: Fig S3). As expected, most strains were clustered with *P. syringae* (PG02b) and *P. cerasi* (PG02a) reference strains. By contrast only the two *P. congelans* (PG02c) of our study were included in the cluster with reference strains of this species. It is worth mentioning this, because no isolation of *P. congelans* (PG02c) has been reported in the previous investigations. The *P. syringae* (PG02b) cluster was very homogeneous, with most sequence similarities > 99.5% (Additional file 1: Table S5). In this group, two strains from Iran (FBF27 & 47) had the same *rpoD* sequence as the type strain. A group of 24 strains shared the same *rpoD* sequence having 99.8% similarity with the type strain: all the 12 IZB strains from Serbia, two strains from Iran (FBF111 & 138) and 10 strains from our study (BE1, 3, E10CA, E11 and 12A, EL1A, 2, TRR12, 15 and 19). Finally, remaining 18 *P. syringae* (PG02b) strains from our study and two strains from Iran (FBF46 & 63) exhibited high sequence similarity (99.5%) with the *P. syringae* type strain with this percentage being a bit lower for strains KC46 (99.3%) and E93A (97.9%) (Additional file 1: Table S5).

## Discussion

The increase in economic losses associated with citrus blast and black pit drew our attention to the problem a few years ago, leading to a thorough investigation of the causative agent. There are several aspects to discuss based on the obtained results.

### Spreading of the disease in the center of Tunisia and on various citrus cultivars

The sampling on citrus was performed over two successive seasons during two years to cover all citrus growing regions. In Tunisia, northern citrus–growing regions are known to be particularly affected by the disease. The Cap Bon area including Nabeul governate is the main producing region of citrus and the majority of orchards in this country (24) were selected. Other producing areas selected were Beja, Ben Arous, Bizerte and Jendouba governates situated in the north of Tunisia and the Kairouan governate situated in the south compared to the other places. The orchards were chosen arbitrarily. It may explain the fact of not falling systematically on orchards with lot of symptomatic trees. The low incidence of disease measured did not mean the absence of the disease in the area. Except the Jendouba governate where no symptom was noted, in all other governates it was possible to find symptoms including Kairouan.

In a previous work during the 2012–2015 seasons, Abdellatif et al. (2017) found symptomatic trees in orchards situated in Nabeul, Beja, Ben Arous and Bizerte governates whereas they did not find symptoms in the Kairouan governate. They considered at this time the region of Kairouan still free of the disease because it is located in the northern desert of Tunisia. The climate of this region is characterized by low humidity and high temperature compared to other regions which could explain the healthy situation of citrus orchards (Abdellatif et al. 2017). From the present work, it is clear that the disease has now spread to the Kairouan governate, at least in the Sbikha region, despite these supposed unfavourable climatic conditions (Additional file 1: Table S1). It shows the pressure of the disease is sufficiently high to contaminate a region with an unfavorable climate.

During sampling campaigns, symptoms were observed mainly on fruits, especially in spring when the delay in the harvest period coincides with higher temperature and humidity, favorable environmental conditions to disease development. Abdellatif et al. (2017) found also the disease principally as black pit on fruits and sometimes as blast on twigs, on 5 various citrus cultivars. Nevertheless isolation of pathogenic strains was successful only in 10 out 24 symptomatic orchards and among the 45 pathogenic *Pseudomonas* sp. isolated, 44 were from *C. limon* cv. 'Eurêka' the last being isolated from *C. sinensis* cv.’Washington Navel’. In the present work successful isolation was also low (positive in 10 out 37 orchards) and probably due to the low level of bacterial population or its migration in another part of the plant. The disease was confirmed on C*. limon* cv. ‘Eurêka’ and its spread to many other cultivars was also shown, taking into account all orchards, eight cultivars developed symptoms (Additional file 1: Table S1). Pathogenic *Pseudomonas* was isolated from 6 of them: *C. limon* cvs. ‘Eurêka’ and ‘Lunari’, *C. reticulata* cv. Hernandina’ and ‘Cassar’ and *C. sinensis* cvs. ‘Maltaise’ and ‘Valencia Late’. It shows that the disease spread geographically as well as to more and more citrus cultivars.

### Diversity of strains from the *P. syringae* group of bacteria

In the present study, among the 820 strains isolated on KB, 41 strains (5%) were identified to the *P. syringae* group of bacteria. They all belong to the PG02 phylogroup following the current *P. syringae* classification proposed by Berge et al. (2014). The *P. syringae* PG02 phylogroup is ubiquitous, it contains many pathogenic strains isolated from a wide range of plants and could be isolated from very diverse subtsrates (wild plant, river, snow, rain …). It contains mostly very aggressive strains when tested on cantaloupe seedlings, frequent ice nucleation active bacteria and strains able to produce a syringomycin- like toxin (Berge et al. 2014). *P. syringae* PG02 strains of this study were identified to three species. As shown previously, the most prevalent on citrus was the *P. syringae* PG02b that includes the *P. syringae* pv. *syringae* van Hall 1902, PDDCC 3023^ T^ type strain. The majority of isolated strains in this work (75%, 33 strains) were affiliated with this species. They were isolated in many places and cultivars. They were found on 5 citrus cultivars in 8 orchards located in 4 governates. This study confirmed that these strains play a major role in the disease on citrus in Tunisia as shown previously (Abdellatif et al. 2017).

The intragroup diversity of our *P. syringae* PG02b strains was explored using BOX-PCR fingerprinting and it revealed that these strains were diverse, exhibiting at least 7 different fingerprints (Table 2). However, one clonal strain (P2 BOX-profile) was more frequent and it could represent a dominant epidemic strain widespread in Tunisia. A quantitative approach during isolation could help to know better the real prevalence of each type of strain and their involvement in the diseases. In addition to the expected *P. syringae* (PG02b) and for the first time in Tunisia, 6 strains from the *P. syringae* PG02, identified as *P. cerasi* (*P. syringae* PG02a) were found. They represented 14% of isolated strains and were found in only one orchard classified in class1 of sanitary state, on *C. sinensis* ‘Maltaise’ independently of the PG02b strains. This work demonstrated that these strains could produce characteristic symptoms of the diseases in Tunisia, likely with a lower level of aggressiveness than *P. syringae* (PG02b). For some of them they were very aggressive on cantaloupe and could be involved in outbreaks of such crops if grown near citrus orchards (Table 3). The species *P. cerasi* is closely related to *P. syringae* (PG02b) and was first described with strains isolated from diseased tissue of cherry trees in Poland (Kaluzna et al. 2016). However, this species potentially included strains of the PG02a phylogroup, isolated from wild plant (Hirano and Upper 1990), rain and irrigation basin (Berge et al. 2014).

The presence of this species in citrus diseases and its spreading in other regions and cultivars need to be studied in the future in Tunisia in order to better understand its role in the disease. The last species of the *P. syringae* PG02 was *P. congelans* (*P. syringae* PG02c) with 4% of isolated strains (2 strains) from 2 orchards located in 2 governates. *P. congelans* (PG02c) strains were isolated from *C. reticulata* 'Hernandina' and *C. sinensis* 'Valencia Late', presenting the gummosis of the branches and the black pit respectively. One of these two strains was tested non- pathogenic on citrus, but was very aggressive on cantaloupe and positive in the HR test. The strains were present on trees of sanitary status classified as class 2 and 3. *P. congelans* was first described as non-pathogenic from the phyllosphere of grasses (Behrendt et al. 2003) and actually potentially included strains of the PG02c phylogroup. These strains have been described previously as dominated by pathogenic strains isolated sometimes from plants and frequently from environmental substrates linked to the water cycle (Berge et al. 2014) or having a biocontrol activity against fire blight (Mikicińskiet al. 2020). At the opposite of the other strains from the *P. syringae* phylogroup PG02 having the canonical Type Three Secretion System (T3SS) used to secrete proteins that help the bacteria infect plant cells, PG02c strains have an atypical T3SS similar to S-PAI of *P. viridiflava* (Clarke et al. 2010). However, these strains can be pathogenic (Demba Diallo et al. 2012). Both *P. congelans* (PG02c) strains were resistant to streptomycin, the main antibiotic currently in use for plant disease control as well as to chloramphenicol, amoxcillin, tobramycin and carbenicillin. One of these strains was classified as a Multi Drug Resistant organism in our tests (Additional file 1: Table S3). It seems that this group of bacteria is not directly involved in the citrus diseases: however, it could play a role for example during late frost, through its ice nucleation activity tested at − 1.5 °C. Thus, the pathogenic population could more easily colonize the frozen tissues damaged by ice crystals produced by *P. congelans*.

Koch’s postulates were validated on citrus for more than one epidemic strain of *P. syringae*. It is clear from this work that bacteria from at least two species, *P. syringae* (PG02b) and *P. cerasi* (PG02a) were able to induce symptoms on citrus and may be involved in the diseases. Moreover, the isolated strains were diverse and more than 7 BOX-profiles were found showing the non-clonality of the pathogenic strains. The citrus-*P. syringae* pathosystem is complex as shown previously on *Prunus* species. Ruinelli et al. (2019) found that a large number of various *P. syringae* strains are pathogenic on the tested hosts (cherry, peach and almond). Similarly Parisi et al. (2019), isolated four phylogroups (PG01, 02, 03 and 07) able to induce canker on apricot. Ruinelli et al. (2019) suggest the diversity of strains pathogenic on *Prunus* is probably due to independent evolution of individual strains, not necessarily related to virulence.

### Diverse other *Pseudomonas* species isolated from symptomatic trees

Beyond tracking the *P. syringae* group of bacteria, this study aimed to prospect for the population diversity of some other *Pseudomonas* species associated with the disease, following the work of Beiki et al. (2016). They showed that a very diverse population of *Pseudomonas* strains was associated with citrus blast in Iran: some from the *P. syringae* group of bacteria as expected but also and for the first time, other species such as *P. lurida, P. monteilli, P. moraviensis, P. orientalis*, *P. simiae*, and the new species *P. caspiana* (Busquets et al. 2017). To reach this goal, 6 strains of *P. moraviensis* were isolated coexisting with *P. syringae* strains from *C. sinensis* cv. 'Valencia Late' and *C. limon* cv. ‘Lunari’ fruits. The other 4 *P. moraviensis* strains were isolated independently from *P. syringae* on *C. limon* cv. 'Eurêka'. All trees were classified in the class 3 sanitary state (Additional file 1: Table S1). Only one strain was tested for pathogeny and was negative. *P. moraviensis* (Tvrzovà et al. 2006) is an ubiquist species with high metabolic versatility and bioremediation potential (Miller et al. 2016). It is also considered as a PGPR (Plant Growth-Promoting Rhizobacteria) (Hassan et al. 2016). Two other *Pseudomonas* species, *P. lactis* and *P. oryzihabitans* were isolated respectively from *C. reticulata* cv.’Hernandina’ and *C. sinensis* cv. ‘Valencia Late’ coexisting with some *P. syringae* strains on trees classified as class 2 sanitary status (Additional file 1: Table S1). In citrus orchards, these three *Pseudomonas* species not closely related to the *P. syringae* group of bacteria (Mulet et al. 2010) should be considered as opportunistic bacteria associated with symptoms (Beiki et al. 2016) and their involvement in the disease needs to be explored. In the same survey as this study, two new species pathogenic on citrus *P. kairouanensis* and *P. nabeulensis* formerly described elsewhere (Oueslati et al. 2019), were found respectively in orchards surveyed in Kairouan and Nabeul governates (Additional file 1: Table S1). These new citrus pathogens may be involved in the disease as well as the *P. syringae* group of bacteria.

### Diversity of *P. syringae* in the Mediterranean area and Caspian regions

It has been demonstrated previously in different countries that citrus blast and citrus black pit are caused primarily by homogenous populations of *P. syringae* (PG02b) close to the type strain of *P. syringae* pv. *syringae* PDDCC 3023^ T^ like in Turkey (Mirik et al. 2005). Abdellatif et al. (2017) suggested the homogeneity of *P. syringae* strains isolated in Tunisia on citrus might have originated from a single inoculum source linked to commercial exchanges following Ivanović et al. (2017) in Montenegro. The latter demonstrated that the single strain of *P. syringae* PG02b, causing citrus blast on mandarin in this country, originated from planting material. In Iran, Beiki et al. (2016) isolated some *P. syringae* (PG02b) strains close to the type strain PDDCC 3023^ T^ together with very diverse pathogenic *Pseudomonas* species involved in the disease.

The phylogenetic position of our strains was compared with those from Iran and from Serbia using *rpoD* gene sequences. It was not possible to include strains previously isolated in Tunisia, because they were identified using 16S rRNA gene sequences (Abdellatif et al. 2017) unsuitable to discriminate between *Pseudomonas* species, neither phylogroups of the *P. syringae* group of bacteria (Yamamoto et al. 2000; Parkinson et al. 2011). Based on *rpoD* phylogeny, it is clear that a group of closely related strains from *P. syringae* (PG02b) is involved in citrus diseases in the three different countries. These strains are not clonal, as far as they represented various *rpoD* haplotypes (Additional file 1: Fig. S3), but probably shared the main phenotypic traits linked to pathogeny. The wide dispersion of this group of *P. syringae* (PG02b) in the Mediterranean Basin and Caspian region and the high similarities in the *rpoD* nucleotide sequences suggest they are a group of strains well-adapted (high fitness) to citrus. Some strains affiliated with the PG02d clade were isolated in Iran, not all are pathogenic on citrus (Beiki et al. 2016). This clade is very closely related to the *P. syringae* PG02b and was not found in Tunisian orchards. Another large group of related strains involved in the disease in two of the three countries appears clearly in Additional file 1: Fig. S3 as being the *P. cerasi* (PG02a) species. These strains are not clonal and may be more variable among phenotypes (Additional file 1: Table S4). This group represents an emerging pathogen in Tunisia, where it was found in only one orchard. Monitoring their spreading in other regions and cultivars in the future could help to see their fitness in this context. Exploring their phenotypic and genotypic structures and their pathogenic characteristics could help to better understand their role in the disease.

Finally, for the first time a third group of strains belonging to *P. congelans* (PG02c) was isolated on citrus. Although not pathogenic on citrus, they can be very aggressive on cantaloupe, and active for ice nucleation at high temperature (− 1.5 °C). It would be interesting to explore further their role in the citrus diseases. This species had not been described on citrus so far, because it does not produce symptoms on plant tissues when inoculated in pure culture. This *P. congelans* (PG02c) bacteria could be tested in co-inoculation experiments with a *P. syringae* pathogenic strain to see the impact of this bacterium on symptom development particularly if doing a cold shock.

In conclusion, this study demonstrated that citrus diseases have progressed in Tunisia. The governate of Kairouan is now affected by the disease despite their non-favorable climate. The pathogenic strain affiliated to *P. syringae* (PG02b) previously described on citrus were frequently isolated in Tunisian orchards and spread in Tunisia in all regions and on many cultivars. This species is widely distributed in producing countries and may represent an epidemic group of strains disseminated through plant material or commercial exchanges. Moreover, our work showed that this pathogen is not unique. One more pathogenic species of the *P. syringae* group, *P. cerasi* (PG02a) and two new pathogenic species of *Pseudomonas*, *P. kairouanensi*s and *P. nabeulensis* are involved in the diseases in Tunisia (Oueslati et al. 2019). *P. cerasi* (PG02a) is emergent in Tunisia and requires special attention as it was frequently isolated previously in Iran and was aggressive on citrus (Beiki et al. 2016). It could disseminate and cause damages and economic losses. In the same way, the two new described pathogenic species were isolated only in one orchard and little information is available on them and the danger they represent. *P. cerasi* (PG02a), *P. kairouanensis* and *P. nabeulensis*, should be included in future surveys of pathogens involved in citrus blast and black pit in Tunisia as well as in other countries where these diseases are present. It will be important to include these species in the diagnostic scheme, identification of reservoirs (Mougou and Boughalleb-M'hamdi 2016) and the biocontrol research mainly focused on *P. syringae* (PG02b) (Mougou and Boughalleb-M’hamdi 2016).

To be effective and to have an adequate biocontrol measures using antagonistic bacteria or bacteriophages (Braun-Kiewnick et al. 2000; Pinheiro et al. 2019), it is then necessary to know very well the pathogenic populations responsible for the disease. For that, isolation of pathogenic bacteria using an approach without a priori is particularly efficient when ubiquitous diverse bacteria like those of the *P. syringae* group of bacteria are involved. In addition to their ability to disseminate between cultivars and sites, Vasebi et al. (2019) have shown that strains of *P. syringae* pv. *syringae* isolated from diseased apricot trees can adapt to other alternative hosts such as citrus and cause symptoms. The polyphagous nature of these bacteria allows it to pass from one crop to another and requires the use of an integrated management targeting several cultures in a region. Our results from the phenotypic and genomic variability of *P. syringae* can be used for studies on varietal susceptibility, the determination of the host range and the development of management and control strategies for this topical bacterial disease. Finally, the presence of ubiquitous species in symptomatic tissue like *P. congelans*, *P. moraviensis, P. lactis and P. oryzyhabitans* and some others open new perspectives of studies on their role in the development of the disease and their interactions with pathogens.

This study reveals that this disease is more complex than previously thought and pathogenic bacteria involved are diverse and complex. Disease on trees can develop during more than only one season and different part of the plant and various pathogens can succeed each other, or cohabited and cooperate. The economic context and the pressure of the market on the citrus growers led to a longer and longer delay of the harvest that favors the dissemination and growth of bacterial populations and the development of symptoms. In that context, to limit the effect of the diseases on yield, it will be very important to determine the role of each pathogen during the season and their interactions with each other and with the plant microbiota.

## Supplementary information

**Additional file 1: Table S1.** Localization and characteristics of the Tunisian orchards surveyed for blast and black pit disease in 2015, 2016 and 2017. **Table S2.** Other *Pseudomonas* strains characteristics isolated from symptomatic samples. **Table S3.** Detection of enzymatic activity of *P. syringae* and *P. congelans* strains of this study, using API ZYM system. **Table S4.** Resistance antibiotic patterns of the *P. syringae* and *P. congelans* strains used in this study. **Table S5.** Matrix of pairwise genetic similarity of *rpoD* gene sequences of strains of this study, strains from symptomatic citrus in Iran (FBF strains) and in Serbia (IZB strains). **Fig. S1.** Phylogenetic tree built with Neighbor joining method based on *cts* partial sequences of *P. syringae* strains isolated from Tunisian citrus orchards. **Fig. S2.** BOX fingerprints of representative strains of the *P. syringae* group isolated from citrus in symptomatic Tunisian orchards from different regions. **Fig. S3.** Phylogenetic tree based on the *rpoD* gene sequences of *P. syringae* strains of this study, together with other strains of *P. syringae* from Tunisia, (Abdellatif et al. 2017), Montenegro (Ivanović et al. 2017) and Iran (Beiki et al. 2016). Distance matrices were calculated by the Jukes-Cantor method (Jukes and Cantor 1969). Dendrograms were generated by the neighbour-joining method. *P. aeruginosa* ATCC 10145 T was used as the outgroup. The bar indicates sequence divergence. Percentage bootstrap values of more than 50% (from 1000 replicates) are indicated at the nodes. GenBank accession numbers are given in parentheses.
