# Supplementary material for: Diversity of pathogenic Pseudomonas isolated from citrus in Tunisia
Source: AMB Express. 2020 Nov 1;10:198. doi: 10.1186/s13568-020-01134-z (PMC7604283; doi:10.1186/s13568-020-01134-z)
Supplement: Supplementary file 1 — Additional file 1: Table S1. Localization and characteristics of the Tunisian orchards surveyed for blast and black pit disease in 2015, 2016 and 2017. Table S2. Other Pseudomonas strains characteristics isolated from symptomatic samples. Table S3. Detection of enzymatic activity of P. syringae and P. congelans strains of this study, using API ZYM system. Table S4. Resistance antibiotic patterns of the P. syringae and P. congelans strains used in this study. Table S5. Matrix of pairwise genetic similarity of rpoD gene sequences of strains of this study, strains from symptomatic citrus in Iran (FBF strains) and in Serbia (IZB strains). Fig. S1. Phylogenetic tree built with Neighbor joining method based on cts partial sequences of P. syringae strains isolated from Tunisian citrus orchards. Fig. S2. BOX fingerprints of representative strains of the P. syringae group isolated from citrus in symptomatic Tunisian orchards from different regions. Fig. S3. Phylogenetic tree based on the rpoD gene sequences of P. syringae strains of this study, together with other strains of P. syringae from Tunisia, (Abdellatif et al. 2017), Montenegro (Ivanović et al. 2017) and Iran (Beiki et al. 2016). Distance matrices were calculated by the Jukes-Cantor method (Jukes and Cantor 1969). Dendrograms were generated by the neighbour-joining method. P. aeruginosa ATCC 10145 T was used as the outgroup. The bar indicates sequence divergence. Percentage bootstrap values of more than 50% (from 1000 replicates) are indicated at the nodes. GenBank accession numbers are given in parentheses. [file 13568_2020_1134_MOESM1_ESM.pdf]

AMB Express

Diversity of pathogenic *Pseudomonas* isolated from citrus in Tunisia

Oueslati Maroua[1], Magdalena Mulet[2], Zouaoui Mohamed[1], Chandeysson Charlotte[3], Jorge Lalucat [2,4], Mohamed Rabeh Hajlaoui[5], Odile Berge[3], Elena García-Valdés[2,4], Najla Sadfi -Zouaoui[1]

[1]Laboratoire de Mycologie, Pathologies et Biomarqueurs (LR16ES05), Département de Biologie, Université de Tunis-El Manar, 2092 Tunis, Tunisie

[2]Microbiologia, Departament de Biologia, Edifici Guillem Colom, Universitat de les Illes Balears, Campus UIB, 07122 Palma de Mallorca, Spain

[3]INRAE, Pathologie Végétale F-84140, Monfavet, France

[4]Institut Mediterrani d'Estudis Avançats (IMEDEA, CSIC-UIB), Campus UIB, 07122 Palma de Mallorca, Spain

[5]Laboratoire de Biotechnologie Appliquée à l'Agriculture, INRA Tunisia, Université de Carthage, 2094 Ariana, Tunisia

Corresponding author: Sadfi.Najla@gmail.com

Telephone: (+216) 71872 600

Fax: (+216) 71885 480

Supplementary Table 1 Localization and characteristics of the Tunisian orchards surveyed for blast and black pit disease in 2015, 2016 and 2017

Governorate	Region	Orchard name	GPS localization	Orchard type[a]	Average annual precipitation (mm)	Average annual temperature (°C)	Year of planting	Irrigation system	Type of culture	Sanitary state[c]	Year of sampling	Cultivars sampled	Symptoms	Percentage of symptomatic trees / orchards	Symptomatic trees analysed / orchard	Number of sampled studied	Number of samples with successful isolation of *P. syringae* putative strains[d]	Total Number of strains isolated	Total Number of strains charcterized in this paper	Strains rapidly identified to the *P. syringae* group before to be lost, and reported in this study[e]	Strains deeply identified to the *P. syringae* group, reported in this study	Other strains of *Pseudomonas* sp. deeply identified, reported in this study	Strains previously identified as new Citrus pathogenic species (Oueslati et *al* . 2019)
Nabeul	Bni khalled	Technical Center of Citrus (TCC)	36.630202, 10.558902	Mixed	400	17.7	1990	Drip irrigation	Conventional	class 3	2015	ND	No Symptom	0	o	0	0	0	0	0	0	0	0
Nabeul	Bni khalled	Technical Center of Citrus (TCC)	36.630202, 10.558902	Mixed	400	17.7	1990	Drip irrigation	Conventional	class 3	2016	*C. limon* 'Eurêka'; *C. reticulata 'Cassar'*	Black pit	2.2	1	14	7	26	7	5 *P. syringae* PG02b	2 *P. syringae* PG02b	0	0
Nabeul	Bni khalled	Dhaouadi	36.661788, 10.587788	Mixed	400	17.7	1982	Surface irrigation	Conventional	class 3	2016	*C. limon* 'Eurêka'	Blast	0.5	1	11	2	24	3	0	3 *P. syringae* PG02b	0	0
Nabeul	Bni khalled	Snoussi	36.629742, 10.582372	Mixed	440	17.8	1962	Surface irrigation	Conventional	class 2	2015	ND	No Symptom	0	0	0	0	0	0	0	0	0	0
Nabeul	Bni khalled	Chakib	36.649773, 10.587161	Mixed	400	17.7	1982	Surface irrigation	Conventional	class 2	2016	ND	No Symptom	0	0	0	0	0	0	0	0	0	0
Nabeul	Bni khalled	Chakib	36.649773, 10.587161	Mixed	400	17.7	1982	Surface irrigation	Conventional	class 2	2017	*C. sinensis* 'Maltaise '; *C. limon* 'Eurêka'	Black pit; Blast	5.0	3	14	3	34	5	0	5 *P. syringae* PG02b	0	1 (2016) & 3 (2017) *P. nabeulensis*
Nabeul	Bni khalled	Kawaris	36.613761, 10.570699	Mixed	400	17.7	1975	Drip irrigation	Conventional	class 2	2016	*C. paradisi* 'Star ruby'	Black pit	2.2	1	2	0	3	0	0	0	0	0
Nabeul	Bni khalled	Bosten	36.648475, 10.548040	Mixed	351	17.7	1985	Drip irrigation	Conventional	class 1	2016	*C. sinensis* 'Maltaise '	Black pit	2.5	3	6	0	18	0	0	0	0	0
Nabeul	Bni khalled	Soussi	36.634702, 10.548040	Mixed	351	17.7	1996	Surface irrigation	Conventional	class 2	2016	*C. paradisi* 'Star ruby'	Black pit	1.0	1	2	0	3	0	0	0	0	0
Nabeul	Grombelia	Housaini	36.580409, 10.537054	Mixed	423	17.6	1996	Surface irrigation	Conventional	class 2	2016	*C. sinensis* 'Maltaise '	Black pit	1.5	2	4	0	16	0	0	0	0	0
Nabeul	Grombelia	Hwas	36.624507, 10.501348	Mixed	423	17.6	1989	Surface irrigation	Conventional	class 1	2015	*C. limon* 'Eurêka'	Black pit	ND	1	1	0	8	0	0	0	0	0
Nabeul	Tekelsa	SOTAM	36.801510, 10.644448	Mixed	397	17.7	1990	Drip irrigation	Conventional	class 2	2015	*C. reticulata* 'Hernandina'	Blast; Gummosis	0.8	1	2	1	14	3	0	1 *P. congelans* (*P. s.* PG02c)	2 *P. lactis*	0
Nabeul	Tekelsa	Mraisa	36.792952, 10.660650	Mixed	397	17.7	1980	Drip irrigation	Organic	class 3	2015	*C. sinensis* 'Maltaise '	Gummosis	ND	2	3	0	8	0	0	0	0	0
Nabeul	Bouargoub	Torki	36.528560, 10.589239	Mixed	440	17.8	ND[b]	Surface irrigation	Organic	class 2	2015	*C. reticulata* 'Hernandina'; *C. sinensis* 'Valencia Late'	Blast; Gummosis	1.1	4	6	0	20	0	0	0	0	0
Nabeul	Bouargoub	Tlili	36.522491, 10.553533	Homogeneous	415	17.9	1992	Surface irrigation	Conventional	class 3	2017	*C. limon* 'Eurêka'	Black pit	1.2	1	5	2	33	2	0	0	2 *P. moraviensis*	0
Nabeul	Bouargoub	Ziadi	36.592538, 10.546667	Mixed	400	17.7	1991	Surface irrigation	Organic	class 2	2016	*C. limon* 'Eurêka'	Black pit	ND	2	3	0	37	0	0	0	0	0
Nabeul	Bouargoub	ATLAS	36.529664, 10.601598	Mixed	383	17.6	1985	Drip irrigation	Conventional	class 2	2015	ND	No Symptom	0	0	0	0	0	0	0	0	0	0
Nabeul	Bouargoub	Ben Fkhih	36.508695, 10.578253	Mixed	415	17.9	1970	Drip irrigation	Conventional	class 2	2016	ND	No Symptom	0	0	0	0	0	0	0	0	0	0
Nabeul	Bouargoub	Charfeddine	36.553937, 10.645544	Homogeneous	440	17.8	1991	Drip irrigation	Organic	class 1	2016	*C. sinensis* 'Maltaise '	Black pit	3.0	4	8	6	17	6	6 *P. syringae* PG02a (*P. cerasi*)	0	0	0
Nabeul	Bouargoub	The Gardens	36.738452, 10.611013	Mixed	351	17.7	1971	Drip irrigation	Conventional	class 3	2015	*C. limon* 'Lunari'	Black pit	4.4	4	16	4	31	5	0	3 *P. syringae*	2 *P. moraviensis*	0
Nabeul	ElGobba	Eltehem Rhouma	36.806264, 10.940591	Mixed	383	17.6	ND	Drip irrigation	Organic	class 2	2015	*C. sinensis* 'Maltaise '	Black pit	1.4	3	5	0	17	0	0	0	0	0
Nabeul	ElGobba	Amara	36.809563, 10.918618	Homogeneous	400	17.7	1970	Drip irrigation	Conventional	class 2	2015	*C. sinensis* 'Valencia Late'	Black pit	ND	2	11	0	22	0	0	0	0	0
Nabeul	Bouzelfa	Elmachat	36.695129, 10.603448	Mixed	400	17.7	ND	Drip irrigation	Organic	class 3	2015	*C. sinensis* 'Washington Navel'	Blast; Black pit	ND	2	7	0	11	0	0	0	0	0
Nabeul	Bouzelfa	Elloumi	36.702287, 10.606881	Mixed	400	17.7	1990	Drip irrigation	Conventional	class 2	2016	*C. limon* 'Lunari'	Black pit	2.5	3	9	0	14	0	0	0	0	0
Nabeul	Bouzelfa	Khsairi	36.668147, 10.596581	Mixed	400	17.7	1973	Drip irrigation	Conventional	class 2	2015	ND	No Symptom	0	0	0	0	0	0	0	0	0	0
Nabeul	Bouzelfa	Bouchrida	36.681915, 10.558816	Mixed	351	17.7	ND	Drip irrigation	Conventional	class 3	2016	ND	No Symptom	0	0	0	0	0	0	0	0	0	0
Ben Arous	Khlidia	Khlidia	36.647766, 10.334283	Homogeneous	448	18,0	1970	Drip irrigation	Conventional	class 3	2016	*C. limon* 'Lunari'	Black pit	1.25	1	3	0	17	0	0	0	0	0
Ben Arous	Naasen	Ayari	36.696114,10.275442	Mixed	448	18,0	1990	Drip irrigation	Conventional	class 2	2017	*C. limon* ' Eurêka'; *C. sinensis* 'Maltaise'	Black pit	3.5	3	13	3	25	6	0	6 *P. syringae* PG02b	0	0
Ben Arous	Morneg	Morneg 1	36.673103, 10.277291	Mixed	446	18.1	1935	Drip irrigation	Organic	class 3	2016	*C. limon* 'Eurêka'	Black pit	1.7	5	8	0	7	0	0	0	0	0
Ben Arous	Morneg	Morneg 2	36.673103, 10.277291	Mixed	446	18.1	1935	Drip irrigation	Organic	class 3	2016	*C. limon* 'Eurêka'	Black pit	ND	2	5	0	9	0	0	0	0	0
Bizerte	Ras Jbel	Ras Jbel 1	37.207796, 10.059716	Mixed	472	17.7	1988	Surface irrigation	Organic	class 3	2015	*C. limon* 'Eurêka'	Black pit	22.5	9	18	0	32	0	0	0	0	0
Bizerte	Ras Jbel	Ras Jbel 2	37.207796, 10.059716	Mixed	472	17.7	1988	Surface irrigation	Organic	class 3	2015	*C. limon* 'Eurêka'	Black pit	7.5	3	9	0	13	0	0	0	0	0
Bizerte	Ras Jbel	Essghaier	37.213265, 10.032251	Mixed	472	17.7	1990	Drip irrigation	Conventional	class 3	2016	*C. limon* 'Eurêka'	Black pit	ND	6	14	0	28	0	0	0	0	0
Beja	Wechteta	Miledi	36.960478, 8.992101	Mixed	450	18.2	1980	Surface irrigation	Conventional	class 3	2017	*C. sinensis* 'Washington Navel'	Blast	2.2	1	13	1	15	2	0	0	2 *P. moraviensis*	0
Beja	Nefza	Miled Guennouni	36.972154, 9.052573	Mixed	450	18.2	1987	Surface irrigation	Conventional	class 3	2017	*C. sinensis* 'Valencia Late'	Black pit; Blast	5.6	4	25	5	41	7	0	2 *P. syringae* PG02b; 1 *P. congelans* (*P. s.* PG02c)	4 *P. moraviensis*	0
Jendouba	Bouselem	Bouselem 1	36.636905, 9.002282	Mixed	527	18,0	1971	Surface irrigation	Conventional	class 3	2017	ND	No Symptom	0	0	0	0	0	0	0	0	0	0
Jendouba	Bouselem	Bouselem 2	36.619271, 8.924005	Mixed	527	18,0	1971	Surface irrigation	Conventional	class 3	2017	ND	No Symptom	0	0	0	0	0	0	0	0	0	0
Kairouan	Sbikha	Chaabani1	35.850740,10.054324	Homogeneous	293	18.8	1966	Drip irrigation	Organic	class 2	2017	*C. sinensis* 'Valencia Late'	Black pit	0.5	1	13	2	146	1	0	1 *P. syringae* PG02b	0	0
Kairouan	Sbikha	Chaabani2	35.850740,10.054324	Homogeneous	293	18.8	1966	Drip irrigation	Organic	class 2	2017	*C. sinensis* 'Valencia Late'	Black pit	19.0	5	29	5	131	7	0	6 *P. syringae* PG02b	*1 P. orizyhabitans*	7 *P. kairouanensis*
															81	279	41	820	54				

[a] Orchard types: Mixed : mixed *Citrus* species; Homogenous : only one *Citrus* species; Rootstock are C*itrus aurantium* or citrange

[b] ND : Not Determined

[c] Sanitary sate was rated, class 1 (tree without visible symptoms), class 2 (tree with moderate symptoms) or class 3 (tree with large symptoms)

[d] Strains were firstly classified as putative *P. syringae* using colony morphology. Their definitive identification was performed using Maldi-TOF and housekeeping genes phylogenetic analyses (see the text)

e Strains were affiliated in *P. syringae* phylogroups (PG) following Berge et al. 2014. See the text for details on identification and classification.

Supplementary Table 2 Other *Pseudomonas* strains characteristics isolated from symptomatic samples

Strain	Taxonomic position	Aggresssivness on cantaloupe: Mean symptom rate [a]	Production of syringomycin[b]	Necrosis diameter after injection in fruit(cm)[c]
**BE5**	*P. moraviensis*	0.1	0	0.25± 0.28
**BE8**	*P. moraviensis*	ND	ND	0
**BE10**	*P. moraviensis*	ND	ND	0
**BE28**	*P. moraviensis*	ND	ND	0
**E2B3**	*P. moraviensis*	ND	ND	0
**E2B4**	*P. moraviensis*	ND	ND	0
**KC96A**	*P. orizyhabitans*	0.1	0	ND
**MTR3A**	*P. lactis*	ND	ND	ND
**MTR4**	*P.lactis*	0.2	0	ND
**TRR4**	*P. moraviensis*	ND	ND	0
**TRR18**	*P. moraviensis*	ND	ND	0
**WE101A**	*P. moraviensis*	ND	ND	0
**WE101B**	*P. moraviensis*	ND	ND	0
**CC94**	*P. syringae* (PG02d)	2.7	1.2	ND
**KB49**	*P. syringae* (PG02b)	3.5	1.1	2.3±0.4
**Water**	•	0	0	0

[a]Symptom intensity was rated on 12 cantaloupe seedlings from 0 (no symptom) to 4 (dead plantlet) seven days after inoculation

[b]Production of syringomycine-like toxins was tested on agar medium using *Geotrichum albicans* as a test organism

[c]Necrotic diameters on fruits were measured in cm (average of three replicates ± standard deviation)

CC94/ KB49: positive control

Water : negative control , ND : not determined

Supplementary Table 3 Detection of enzymatic activity of *P. syringae* and *P. congelans* strains of this study, using API ZYM system

Strain	BE12A	BE1	BE3	Iy3DA	Iy3EA	Iy3GB	Iy5HA	IyGA	IyGC	KB49	KC19	KC29B2	KC46	KC54	KC55	KC82	TRR12	TRR15	TRR9	MTR3B	E10AA	E10CA	E10CB2	E11	E12A	E91	E93A	E9A	EL1A	EL2
**Species**	Pc[a]	Ps	Ps	Ps	Ps	Ps	Ps	Ps	Ps	Ps	Ps	Ps	Ps	Ps	Ps	Ps	Ps	Ps	Ps	Pc	Ps	Ps	Ps	Ps	Ps	Ps	Ps	Ps	Ps	Ps
**Alkaline phosphatase**	0[b]	0	0	0	0	1	0	0	0	0	0	0	0	0	0	0	0	0	0	0	0	0	0	0	0	1	1	0	0	0
**Trypsin**	0	0	0	0	0	0	0	0	0	1	0	0	0	0	0	0	0	0	0	0	0	0	1	0	1	0	0	0	0	0
**D-galactosidase**	0	0	0	0	1	0	0	0	0	0	0	0	1	1	0	0	0	0	0	0	0	0	0	0	0	0	0	0	0	0
**D-glucosidase**	0	0	0	0	1	1	0	0	0	0	0	0	0	0	0	0	0	0	0	0	0	0	0	0	0	1	0	0	0	0
**D-chymotrypsin**	1	1	1	1	1	1	1	1	0	0	0	1	1	1	1	1	1	1	0	0	1	1	1	1	1	1	1	0	0	1
**Esterase (C4)**	2	2	2	1	1	1	1	1	1	0	0	1	1	1	1	1	0	0	2	0	2	2	1	2	1	1	1	1	1	1
**Esterase Lipase (C8)**	3	2	2	3	3	3	3	3	3	3	2	1	2	3	3	3	3	3	3	3	1	3	3	3	2	2	3	3	3	3
**ß-glucosidase**	3	3	3	3	4	4	3	3	3	3	3	3	3	3	3	3	3	3	3	3	3	3	3	3	3	3	4	4	3	4

[a] Pc: *P. congelans* PG02c; Ps : *P. syringae* PG02b

[b] The readings are performed using the API staining range and the results scored from 0 to 5: 0 corresponds to a negative reaction, 5 to a reaction of maximum intensity and values 1, 2, 3 or 4 are intermediate reactions depending on the level of intensity (3, 4 or 5 being considered as positive reactions). Acid phosphatase, Naphthol-AS-BI-phosphohydrolase and Leucine arylamidase were positive with score = 5, for all strains. ß-galactosidase, ß-glucuronidase, Lipase (C14),Valine arylamidase, Cystine arylamidase, N-acetyl-ß-glucosaminidase, D-mannosidase, D-fucosidase and Control tests were negative (0) for all strains

Supplementary Table 4 Resistance antibiotic patterns of the *P. syringae* and *P. congelans* strains used in this study

Family	Antibiotics	Disk content (µg)	BE12A	BE1	Iy5HA	IyGC	KB49	KC46	KC55	TRR12	TRR15	MTR3B	E10CB2	E12A	E91	EL2	EUCAST clinical Breakpoint Zone diameter (mm)[a]	
			Pc[b]	Ps	Ps	Ps	Ps	Ps	Ps	Ps	Ps	Pc	Ps	Ps	Ps	Ps	S ³	R <
Penicillins	Piperacillin (PIP)	75	19[c]	22	30	20	29	30	35	32	27	29	26	26	26	26	-	-
	Oxacillin (Ox)	1	0 (R)	0 (R)	0 (R)	0 (R)	0 (R)	0 (R)	0 (R)	0 (R)	0 (R)	0 (R)	0 (R)	0 (R)	0 (R)	0 (R)	-	-
Cephalosporins	Ceftazidime (CAZ)	30	39	30	30	29	30	40	33	41	38	47	24	24	44	30	-	-
	Cephalothin (CF)	30	0 (R)	0 (R)	0 (R)	0 (R)	0 (R)	0 (R)	0 (R)	0 (R)	0 (R)	0 (R)	0 (R)	0 (R)	0 (R)	0 (R)	-	-
	Cefazolin (CZ)	30	0 (R)	0 (R)	0 (R)	0 (R)	0 (R)	0 (R)	0 (R)	0 (R)	0 (R)	0 (R)	0 (R)	0 (R)	0 (R)	0 (R)	-	-
	Ceftriaxone (CRO)	30	13	19	20	20	17	17	19	19	22	0 (R)	20	19	12	20	-	-
	Cefuroxime (CXM)	30	0 (R)	0 (R)	0 (R)	0 (R)	0 (R)	0 (R)	0 (R)	0 (R)	0 (R)	0 (R)	0 (R)	0 (R)	0 (R)	0 (R)	-	-
Carbapenems	Imipenem (IMP)	10	50	40 (S)	50 (S)	46 (S)	47 (S)	52 (S)	39 (S)	37 (S)	47 (S)	16 (R)	34 (S)	51 (S)	39 (S)	46 (S)	20	20
Aminopenicillins	Amoxicillin (AMX)	10	0 (R)	24	19	21	16	13	12	15	24	0 (R)	18	19	16	20	-	-
Aminoglycosides	Gentamicin (GM)	10	10	16 (S)	16 (S)	18 (S)	18 (S)	15 (S)	17 (S)	12 (R)	17 (S)	12 (R)	13 (R)	10 (R)	9 (R)	12 (R)	15	15
	Streptomycin (S)	10	0 (R)	18	16	14	20	14	9	13	12	0 (R)	20	13	15	17	-	-
	Neomycin (N)	30	9	19	16	10	14	14	8	16	12	13	5	8	17	15	-	-
	Tobramycin (TM)	85	0 (R)	15	15	15	15	21	14	18	20	0 (R)	26	22	16	18	-	-
	Kanamycin (K)	30	22	21	20	21	29	31	32	29	27	20	15	32	28	26	-	-
Fluoroquinolones	Ciprofloxacin (CIP)	5	15	50 (S)	42 (S)	39 (S)	40 (S)	46 (S)	44 (S)	37 (S)	36 (S)	17 (R)	27 (S)	44 (S)	35 (S)	40 (S)	26	26
	Pefloxacin (PEF)	5	31	32	34	20	32	22	18	30	22	8	19	12	32	30	-	-
	Ofloxacin (OFX)	5	32	28	38	36	32	34	28	33	27	16	20	28	29	36	-	-
Quinolones	Nalidixic acid (Na)	30	24	25	26	22	22	24	19	22	21	0 (R)	18	19	19	25	-	-
Rifampicin (RA)	Rifampicin (RA)	30	19	20	22	24	20	23	23	18	19	16	18	21	18	18	-	-
Sulfonamides	Trimethoprim-Sulfamethoxazole (Co-trimoxazole)(SXT)	1,25	0 (R)	0 (R)	0 (R)	0 (R)	0 (R)	0 (R)	0 (R)	0 (R)	0 (R)	0 (R)	0 (R)	0 (R)	0 (R)	0 (R)	-	-
Polypeptides	Colistin (CL)	50	5	13	10	15	10	15	11	13	12	11	12	12	12	12	-	-
Carboxypenicillin	Carbenicillin (CB)	100	0 (R)	12	10	0 (R)	0 (R)	0 (R)	12	11	19	0 (R)	14	8	5	10	-	-
Monobactams	Aztreonam(ATM)	30	10	17 (S)	12 (R)	16 (R)	18 (S)	13 (R)	9 (R)	14 (R)	7 (R)	30 (S)	23 (S)	15 (R)	13 (R)	16 (R)	18	18
Tetracyclines	Tetracycline (TE)	30	17	30	28	28	30	30	27	29	21	13	23	29	27	28	-	-
Phenicols	Chloramphenicol (C)	30	0 (R)	14	30	25	20	19	19	24	25	0 (R)	20	25	22	20	-	-
Resistance pattern[a]					ATM	ATM		ATM	ATM	GM; ATM	ATM	IMP; GM; CIP	GM	GM; ATM	GM; ATM	GM; ATM		
MDR[a]												MDR3						

[a] Only antimicrobial categories and agents used to define MDR,XDR and PDR for *Pseudomonas* by EUCAST Clinical Breakpoints (http://www.eucast.org)

[b] Pc : *P. congelans* PG02c; Ps : *P. syringae* PG02b

[c] Blotting paper disks, impregnated with the antibiotics, were deposited on nutrient agar , previously inoculated with strains (106 bacteria/ml). After incubation, the zones of inhibition corresponding to a lack of culture were measured using a rule, comparing them with critical values to say that a strain is sensitive (S) or resistant (R)

Supplementary Table 5 Matrix of pairwise genetic similarity of *rpoD* gene sequences of strains of this study, strains from symptomatic citrus in Iran (FBF strains) and in Serbia (IZB strains)

Strains form Iran | Strains form Serbia | Strain from Tunisia isolated in this Study | Reference strains

No.	Strain	P. amygdali LMG 13184T 1	P. avellanae CIP 105176T 2	P. cannabina LMG 5096T 3	P. caricapapayae LMG2152T 4	P. cichorii ATCC 10857T 5	P. congelans LMG 21466T 6	P. ficuserectae CCUG 32779T 7	P. meliae CCUG 51503T 8	P. savastanoi LMG 2209T 9	P. tremae LMG22121T 10	P. viridiflava ATCC 13223T 11	P. asturiensis LPPA 221T 12	P. cerasi LMG 28609T 13	P. caspiana FBF102T 14	P. syringae BE1 15	P. congelans BE12A 16	P. syringae BE3 17	P. syringae E10AA 18	P. syringae E10CA 19	P. syringae E10CB2 20	P. syringae E11 21	P. syringae E12A 22	P. syringae E91 23	P. syringae E93A 24	P. syringae E9A 25	P. syringae EL1A 26	P. syringae EL2 27	P. syringae Iy3DA 28	P. syringae Iy3EA 29	P. syringae Iy3GB 30	P. syringae Iy5HA 31	P. syringae IyGA 32	P. syringae IYGC 33	P. syringae KB49 34	P. syringae KC19 35	P. syringae KC29B2 36	P. syringae KC46 37	P. syringae KC54 38	P. syringae KC55 39	P. syringae KC82 40	P. congelans MTR3B 41	P. syringae TRR12 42	P. syringae TRR15 43	P. syringae TRR9 44	P. aeruginosa ATCC 10145T 45	P. syringae pv. syringae Cit7 PG02a 46	P. syringae pv. syringae PDDCC 3023T PG02b 47	P. syringae pv. lachrymans 508 PG02c 48	P. syringae pv. syringae B728a PG02d 49	Pseudomonas sp. FBF12 50	Pseudomonas sp. FBF13 51	Pseudomonas sp. FBF16 52	P. caspiana FBF24 53	Pseudomonas sp. FBF27 54	Pseudomonas sp. FBF32 55	Pseudomonas sp. FBF33 56	Pseudomonas sp. FBF36 57	Pseudomonas sp. FBF40 58	Pseudomonas sp. FBF46 59	Pseudomonas sp. FBF47 60	Pseudomonas sp. FBF49 61	Pseudomonas sp. FBF52 62	P. caspiana FBF58 63	Pseudomonas sp. FBF60 64	Pseudomonas sp. FBF61 65	Pseudomonas sp. FBF62 66	Pseudomonas sp. FBF63 67	Pseudomonas sp. FBF68 68	Pseudomonas sp. FBF69 69	Pseudomonas sp. FBF71 70	Pseudomonas sp. FBF72 71	Pseudomonas sp. FBF74 72	Pseudomonas sp. FBF77 73	Pseudomonas sp. FBF78 74	Pseudomonas sp. FBF79 75	Pseudomonas sp. FBF82 76	Pseudomonas sp. FBF83 77	Pseudomonas sp. FBF91 78	Pseudomonas sp. FBF97 79	Pseudomonas sp. FBF98 80	Pseudomonas sp. FBF100 81	P. caspiana FBF103 83	Pseudomonas sp. FBF104 84	Pseudomonas sp. FBF106 85	Pseudomonas sp. FBF107 86	Pseudomonas sp. FBF108 87	Pseudomonas sp. FBF109 88	Pseudomonas sp. FBF111 89	Pseudomonas sp. FBF113 90	Pseudomonas sp. FBF115 91	Pseudomonas sp. FBF116 92	Pseudomonas sp. FBF117 93	Pseudomonas sp. FBF118 94	Pseudomonas sp. FBF119 95	P. caspiana FBF122 96	Pseudomonas sp. FBF124 97	Pseudomonas sp. FBF125 98	Pseudomonas sp. FBF134 99	Pseudomonas sp. FBF135 100	Pseudomonas sp. FBF138 101	Pseudomonas sp. FBF139 102	P. syringae pv. syringae IZB200 103	P. syringae pv. syringae IZB201 104	P. syringae pv. syringae IZB204 105	P. syringae pv. syringae IZB208 106	P. syringae pv. syringae IZB209 107	P. syringae pv. syringae IZB212 108	P. syringae pv. syringae IZB214 109	P. syringae pv. syringae IZB218 110	P. syringae pv. syringae IZB219 111	P. syringae pv. syringae IZB221 112	P. syringae pv. syringae IZB228 113	P. syringae pv. syringae IZB231 114
1	P. amygdali LMG 13184T	100																																																																																																																					
2	P. avellanae CIP 105176T	91.8	100																																																																																																																				
3	P. cannabina LMG 5096T	91.0	87.0	100																																																																																																																		
4	P. caricapapayae LMG2152T	94.8	90.5	89.7	100																																																																																																															
5	P. cichorii ATCC 10857T	82.9	81.5	83.1	81.5	100																																																																																																													
6	P. congelans LMG 21466T	93.1	90.3	89.2	92.6	80.6	100																																																																																																											
7	P. ficuserectae CCUG 32779T	98.9	91.6	91.3	95.1	82.6	93.1	100																																																																																																									
8	P. meliae CCUG 51503T	99.1	91.8	91.3	95.3	82.6	93.3	99.3	100																																																																																																						
9	P. savastanoi LMG 2209T	99.5	91.8	90.7	95.3	82.9	93.6	98.9	99.1	100																																																																																																			
10	P. tremae LMG22121T	99.5	91.8	90.7	94.8	83.2	93.1	98.9	99.1	99.5	100																																																																																																	
11	P. viridiflava ATCC 13223T	90.3	88.2	87.6	89.2	82.1	87.9	90.8	90.8	90.3	90.3	100																																																																																														
12	P. asturiensis LPPA 221T	87.1	87.9	84.2	86.8	81.5	85.7	86.8	86.8	87.4	86.8	87.4	100																																																																																													
13	P. cerasi LMG 28609T	93.6	90.0	88.4	92.6	80.9	96.5	93.3	93.6	94.1	93.6	87.6	85.5	100																																																																																												
14	P. caspiana FBF102T	80.6	79.4	81.1	80.3	79.7	81.5	80.9	80.6	80.9	80.6	79.4	77.9	82.1	100																																																																																											
15	P. syringae BE1	93.3	89.5	89.4	92.6	82.1	96.7	93.6	93.8	93.8	93.3	88.7	84.9	97.2	82.9	100																																																																																																					
16	P. congelans BE12A	92.8	89.8	88.4	92.3	79.7	97.9	92.6	92.8	93.3	92.8	88.2	84.9	96.0	80.9	96.0	100																																																																																																				
17	P. syringae BE3	93.3	89.5	89.4	92.6	82.1	96.7	93.6	93.8	93.8	93.3	88.7	84.9	97.2	82.9	100	96.0	100																																																																																																	
18	P. syringae E10AA	93.1	89.2	89.2	92.3	81.8	96.5	93.3	93.6	93.6	93.1	88.4	84.6	97.0	82.6	99.8	95.8	99.8	100																																																																																													
19	P. syringae E10CA	93.3	89.5	89.4	92.6	82.1	96.7	93.6	93.8	93.8	93.3	88.7	84.9	97.2	82.9	100	96.0	100	99.8	100																																																																																											
20	P. syringae E10CB2	93.1	89.2	89.2	92.3	81.8	96.5	93.3	93.6	93.6	93.1	88.4	84.6	97.0	82.6	99.8	95.8	99.8	100	99.8	100																																																																																										
21	P. syringae E11	93.3	89.5	89.4	92.6	82.1	96.7	93.6	93.8	93.8	93.3	88.7	84.9	97.2	82.9	100	96.0	100	99.8	100	99.8	100																																																																																									
22	P. syringae E12A	93.3	89.5	89.4	92.6	82.1	96.7	93.6	93.8	93.8	93.3	88.7	84.9	97.2	82.9	100	96.0	100	99.8	100	99.8	100	100																																																																																								
23	P. syringae E91	93.6	89.2	89.4	92.3	82.4	96.5	93.8	94.1	94.1	93.6	89.0	84.9	97.0	82.6	99.8	95.8	99.8	99.5	99.8	99.5	99.8	99.8	100																																																																																						
24	P. syringae E93A	91.3	87.3	87.3	90.5	80.0	94.8	91.6	91.8	91.8	91.3	86.8	82.6	95.3	80.9	98.2	94.1	98.2	97.9	98.2	97.9	98.2	98.2	97.9	100																																																																																				
25	P. syringae E9A	93.1	89.2	89.2	92.3	81.8	96.5	93.3	93.6	93.6	93.1	88.4	84.6	97.0	82.6	99.8	95.8	99.8	100	99.8	100	99.8	99.8	99.5	97.9	100																																																																																		
26	P. syringae EL1A	93.3	89.5	89.4	92.6	82.1	96.7	93.6	93.8	93.8	93.3	88.7	84.9	97.2	82.9	100	96.0	100	99.8	100	99.8	100	100	99.8	98.2	99.8	100																																																																																	
27	P. syringae EL2	93.3	89.5	89.4	92.6	82.1	96.7	93.6	93.8	93.8	93.3	88.7	84.9	97.2	82.9	100	96.0	100	99.8	100	99.8	100	100	99.8	98.2	99.8	100	100																																																																																
28	P. syringae Iy3DA	93.1	89.2	89.2	92.3	81.8	96.5	93.3	93.6	93.6	93.1	88.4	84.6	97.0	82.6	99.8	95.8	99.8	100	99.8	100	99.8	99.8	99.5	97.9	100	99.8	99.8	100																																																																												
29	P. syringae Iy3EA	93.1	89.2	89.2	92.3	81.8	96.5	93.3	93.6	93.6	93.1	88.4	84.6	97.0	82.6	99.8	95.8	99.8	100	99.8	100	99.8	99.8	99.5	97.9	100	99.8	99.8	100	100																																																																											
30	P. syringae Iy3GB	93.1	89.2	89.2	92.3	81.8	96.5	93.3	93.6	93.6	93.1	88.4	84.6	97.0	82.6	99.8	95.8	99.8	100	99.8	100	99.8	99.8	99.5	97.9	100	99.8	99.8	100	100	100																																																																										
31	P. syringae Iy5HA	93.1	89.2	89.2	92.3	81.8	96.5	93.3	93.6	93.6	93.1	88.4	84.6	97.0	82.6	99.8	95.8	99.8	100	99.8	100	99.8	99.8	99.5	97.9	100	99.8	99.8	100	100	100	100																																																																									
32	P. syringae IyGA	91.1	87.1	87.6	90.3	79.4	94.6	91.3	91.6	91.6	91.1	86.8	82.6	95.1	80.9	97.9	93.8	97.9	98.2	97.9	98.2	97.9	97.9	97.7	97.3	98.2	97.9	97.9	98.2	98.2	98.2	98.2	100																																																																								
33	P. syringae IYGC	93.1	89.2	89.2	92.3	81.8	96.5	93.3	93.6	93.6	93.1	88.4	84.6	97.0	82.6	99.8	95.8	99.8	100	99.8	100	99.8	99.8	99.5	97.9	100	99.8	99.8	100	100	100	100	98.2	100																																																																						
34	P. syringae KB49	93.1	89.2	89.2	92.3	81.8	96.5	93.3	93.6	93.6	93.1	88.4	84.6	97.0	82.6	99.8	95.8	99.8	100	99.8	100	99.8	99.8	99.5	97.9	100	99.8	99.8	100	100	100	100	98.2	100	100																																																																				
35	P. syringae KC19	93.1	89.2	89.2	92.3	81.8	96.5	93.3	93.6	93.6	93.1	88.4	84.6	97.0	82.6	99.8	95.8	99.8	100	99.8	100	99.8	99.8	99.5	97.9	100	99.8	99.8	100	100	100	100	98.2	100	100	100																																																																			
36	P. syringae KC29B2	93.1	89.2	89.2	92.3	81.8	96.5	93.3	93.6	93.6	93.1	88.4	84.6	97.0	82.6	99.8	95.8	99.8	100	99.8	100	99.8	99.8	99.5	97.9	100	99.8	99.8	100	100	100	100	98.2	100	100	100	100																																																																	
37	P. syringae KC46	92.8	89.0	88.9	92.1	81.5	96.3	93.1	93.3	93.3	92.8	88.2	84.4	96.7	82.6	99.5	95.5	99.5	99.8	99.5	99.8	99.5	99.5	99.3	97.7	99.8	99.5	99.5	99.8	99.8	99.8	99.8	97.9	99.8	99.8	99.8	99.8	100																																																															
38	P. syringae KC54	93.1	89.2	89.2	92.3	81.8	96.5	93.3	93.6	93.6	93.1	88.4	84.6	97.0	82.6	99.8	95.8	99.8	100	99.8	100	99.8	99.8	99.5	97.9	100	99.8	99.8	100	100	100	100	98.2	100	100	100	100	99.8	100																																																														
39	P. syringae KC55	93.1	89.2	89.2	92.3	81.8	96.5	93.3	93.6	93.6	93.1	88.4	84.6	97.0	82.6	99.8	95.8	99.8	100	99.8	100	99.8	99.8	99.5	97.9	100	99.8	99.8	100	100	100	100	98.2	100	100	100	100	99.8	100	100																																																												
40	P. syringae KC82	93.1	89.2	89.2	92.3	81.8	96.5	93.3	93.6	93.6	93.1	88.4	84.6	97.0	82.6	99.8	95.8	99.8	100	99.8	100	99.8	99.8	99.5	97.9	100	99.8	99.8	100	100	100	100	98.2	100	100	100	100	99.8	100	100	100																																																										
41	P. congelans MTR3B	92.8	89.8	88.4	92.3	79.7	97.9	92.6	92.8	93.3	92.8	88.2	84.9	96.0	80.9	96.0	100	96.0	95.8	96.0	95.8	96.0	96.0	95.8	94.1	95.8	96.0	96.0	95.8	95.8	95.8	95.8	93.8	95.8	95.8	95.8	95.8	95.5	95.8	95.8	95.8	100																																																												
42	P. syringae TRR12	93.3	89.5	89.4	92.6	82.1	96.7	93.6	93.8	93.8	93.3	88.7	84.9	97.2	82.9	100	96.0	100	99.8	100	99.8	100	100	99.8	98.2	99.8	100	100	99.8	99.8	99.8	99.8	97.9	99.8	99.8	99.8	99.8	99.5	99.8	99.8	99.8	96.0	100																																																										
43	P. syringae TRR15	93.3	89.5	89.4	92.6	82.1	96.7	93.6	93.8	93.8	93.3	88.7	84.9	97.2	82.9	100	96.0	100	99.8	100	99.8	100	100	99.8	98.2	99.8	100	100	99.8	99.8	99.8	99.8	97.9	99.8	99.8	99.8	99.8	99.5	99.8	99.8	99.8	96.0	100	100																																																										
44	P. syringae TRR9	93.3	89.5	89.4	92.6	82.1	96.7	93.6	93.8	93.8	93.3	88.7	84.9	97.2	82.9	100	96.0	100	99.8	100	99.8	100	100	99.8	98.2	99.8	100	100	99.8	99.8	99.8	99.8	97.9	99.8	99.8	99.8	99.8	99.5	99.8	99.8	99.8	96.0	100	100	100																																																									
45	P. aeruginosa ATCC 10145T	61.6	63.1	61.3	60.8	54.8	62.4	60.5	61.2	61.2	61.2	60.8	60.5	62	59.7	61.6	61.6	61.6	61.2	61.6	61.2	61.6	61.6	62	58.4	61.2	61.6	61.6	61.2	61.2	61.2	61.2	59.7	61.2	61.2	61.2	61.2	61.2	61.2	61.2	61.2	61.6	61.6	61.6	61.6	100																																																								
46	P. syringae pv. syringae Cit7 PG02a	93.6	90. 0	88.4	92.6	80.9	96.5	93.3	93.6	94.1	93.6	87.6	85.5	100	82.1	97.2	96.0	97.2	97.0	97.2	97.0	97.2	97.2	97.0	95.3	97.0	97.2	97.2	97.0	97.0	97.0	97.0	95.1	97.0	97.0	97.0	97.0	96.7	97.0	97.0	97.0	96.0	97.2	97.2	97.2	62	100																																																					
47	P. syringae pv. syringae PDDCC 3023T PG02b	93.1	89.2	89.2	92.3	81.8	96.7	93.3	93.6	93.6	93.1	88.4	84.6	97.5	82.6	99.8	96.0	99.8	99.5	99.8	99.5	99.8	99.8	99.5	97.9	99.5	99.8	99.8	99.5	99.5	99.5	99.5	97.9	99.5	99.5	99.5	99.5	99.3	99.5	99.5	99.5	96.0	99.8	99.8	99.8	62	97.5	100																																																			
48	P. syringae pv. lachrymans 508 PG02c	92.6	89.2	87.6	92.1	80.0	97.9	92.3	92.6	93.1	92.6	87.9	84.6	96.0	81.5	96.0	98.6	96.0	95.8	96.0	95.8	96.0	96.0	95.8	94.1	95.8	96.0	96.0	95.8	95.8	95.8	95.8	93.8	95.8	95.8	95.8	95.8	95.5	95.8	95.8	95.8	98.6	96.0	96.0	96.0	62.4	96.0	96.0	100																																																		
49	P. syringae pv. syringae B728a PG02d	93.8	90.5	88.9	93.3	81.2	97.2	93.6	93.8	94.3	93.8	87.9	85.2	98.4	82.4	97.9	97.2	97.9	97.7	97.9	97.7	97.9	97.9	97.7	96.0	97.7	97.9	97.9	97.7	97.7	97.7	97.7	95.8	97.7	97.7	97.7	97.7	97.5	97.7	97.7	97.7	97.2	97.9	97.9	97.9	62.4	98.4	98.2	97.2	100																																																	
50	Pseudomonas sp. FBF12	92.3	89.8	88.6	91.8	80.6	95.3	92.1	92.3	92.8	92.3	86.8	84.6	98.9	81.2	96.0	95.3	96.0	95.8	96.0	95.8	96.0	96.0	95.8	94.1	95.8	96.0	96.0	95.8	95.8	95.8	95.8	93.8	95.8	95.8	95.8	95.8	95.5	95.8	95.8	95.8	95.3	96.0	96.0	96.0	61.2	98.9	96.3	95.3	97.7	100																																															
51	Pseudomonas sp. FBF13	92.6	89.5	87.6	92.1	80.6	96.3	92.3	92.6	93.1	92.6	86.6	85.2	98.9	81.8	96.3	95.5	96.3	96.0	96.3	96.0	96.3	96.3	96.0	94.3	96.0	96.3	96.3	96.0	96.0	96.0	96.0	94.1	96.0	96.0	96.0	96.0	95.8	96.0	96.0	96.0	95.5	96.3	96.3	96.3	62.4	98.9	96.5	95.5	97.5	97.7	100																																														
52	Pseudomonas sp. FBF16	93.6	90.0	88.4	92.6	80.9	96.5	93.3	93.6	94.1	93.6	87.6	85.5	100	82.1	97.2	96.0	97.2	97.0	97.2	97.0	97.2	97.2	97.0	95.3	97.0	97.2	97.2	97.0	97.0	97.0	97.0	95.1	97.0	97.0	97.0	97.0	96.7	97.0	97.0	97.0	96.0	97.2	97.2	97.2	62	100	97.5	96.0	98.4	98.9	98.9	100																																													
53	P. caspiana FBF24	80.6	79.4	81.1	80.3	79.7	81.5	80.9	80.6	80.9	80.6	79.4	77.9	82.1	100	82.9	80.9	82.9	82.6	82.9	82.6	82.9	82.9	82.6	80.9	82.6	82.9	82.9	82.6	82.6	82.6	82.6	80.9	82.6	82.6	82.6	82.6	82.6	82.6	82.6	82.6	80.9	82.9	82.9	82.9	59.7	82.1	82.6	81.5	82.4	81.2	81.8	82.1	100																																												
54	Pseudomonas sp. FBF27	93.1	89.2	89.2	92.3	81.8	96.7	93.3	93.6	93.6	93.1	88.4	84.6	97.5	82.6	99.8	96.0	99.8	99.5	99.8	99.5	99.8	99.8	99.5	97.9	99.5	99.8	99.8	99.5	99.5	99.5	99.5	97.7	99.5	99.5	99.5	99.5	99.3	99.5	99.5	99.5	96.0	99.8	99.8	99.8	62	97.5	100	96.0	98.2	96.3	96.5	97.5	82.6	100																																										
55	Pseudomonas sp. FBF32	92.6	90.0	88.4	92.1	80.3	95.5	92.3	92.6	93.1	92.6	87.1	84.9	99.1	81.5	96.3	95.5	96.3	96.0	96.3	96.0	96.3	96.3	96.0	94.3	96.0	96.3	96.3	96.0	96.0	96.0	96.0	94.1	96.0	96.0	96.0	96.0	95.8	96.0	96.0	96.0	95.5	96.3	96.3	96.3	61.6	99.1	96.5	95.5	97.9	99.8	97.9	99.1	81.5	96.5	100																																									
56	Pseudomonas sp. FBF33	93.3	90.3	88.1	92.8	80.6	96.7	93.1	93.3	93.8	93.3	87.4	85.7	99.8	81.8	97.0	95.8	97.0	96.7	97.0	96.7	97.0	97.0	96.7	95.0	96.7	97.0	97.0	96.7	96.7	96.7	96.7	94.8	96.7	96.7	96.7	96.7	96.5	96.7	96.7	96.7	95.8	97.0	97.0	97.0	62.4	99.8	97.2	95.8	98.2	98.6	99.1	99.8	81.8	97.2	98.9	100																																								
57	Pseudomonas sp. FBF36	93.6	90.0	88.4	92.6	80.9	96.5	93.3	93.6	94.1	93.6	87.6	85.5	100	82.1	97.2	96.0	97.2	97.0	97.2	97.0	97.2	97.2	97.0	95.3	97.0	97.2	97.2	97.0	97.0	97.0	97.0	95.1	97.0	97.0	97.0	97.0	96.7	97.0	97.0	97.0	96.0	97.2	97.2	97.2	62	100	97.5	96.0	98.4	98.9	98.9	100	82.1	97.5	99.1	99.8	100																																							
58	Pseudomonas sp. FBF40	93.3	89.8	88.1	92.3	80.6	96.7	93.1	93.3	93.8	93.3	87.9	85.7	99.8	81.8	97.0	96.3	97.0	96.7	97.0	96.7	97.0	97.0	96.7	95.0	96.7	97.0	97.0	96.7	96.7	96.7	96.7	94.8	96.7	96.7	96.7	96.7	96.5	96.7	96.7	96.7	96.3	97.0	97.0	97.0	62	99.8	97.2	96.3	98.2	98.6	98.6	99.8	81.8	97.2	98.9	99.5	99.8	100																																						
59	Pseudomonas sp. FBF46	93.1	89.2	89.7	92.3	81.8	96.5	93.3	93.6	93.6	93.1	88.4	84.6	97.0	82.6	99.8	95.8	99.8	99.5	99.8	99.5	99.8	99.8	99.5	97.9	99.5	99.8	99.8	99.5	99.5	99.5	99.5	97.7	99.5	99.5	99.5	99.5	99.3	99.5	99.5	99.5	95.8	99.8	99.8	99.8	61.6	97.0	99.5	95.8	97.7	95.8	96.0	97.0	82.6	99.5	96.0	96.7	97.0	96.7	100																																					
60	Pseudomonas sp. FBF47	93.1	89.2	89.2	92.3	81.8	96.7	93.3	93.6	93.6	93.1	88.4	84.6	97.5	82.6	99.8	96.0	99.8	99.5	99.8	99.5	99.8	99.8	99.5	97.9	99.5	99.8	99.8	99.5	99.5	99.5	99.5	97.7	99.5	99.5	99.5	99.5	99.3	99.5	99.5	99.5	96.0	99.8	99.8	99.8	62	97.5	100	96.0	98.2	96.3	96.5	97.5	82.6	100	96.5	97.2	97.5	97.2	99.5	100																																				
61	Pseudomonas sp. FBF49	93.1	89.5	87.8	92.1	80.6	96.0	92.8	93.1	93.6	93.1	87.1	85.2	99.5	82.1	96.7	95.8	96.7	96.5	96.7	96.5	96.7	96.7	96.5	94.8	96.5	96.7	96.7	96.5	96.5	96.5	96.5	94.6	96.5	96.5	96.5	96.5	96.3	96.5	96.5	96.5	95.8	96.7	96.7	96.7	61.6	99.5	97.0	95.5	97.9	98.4	98.4	99.5	82.1	97.0	98.6	99.3	99.5	99.3	96.5	97.0	100																																			
62	Pseudomonas sp. FBF52	88.2	86.0	86.2	86.8	81.8	85.7	88.7	88.7	88.2	88.2	97.2	87.1	85.5	78.8	86.0	86.0	86.0	85.7	86.0	85.7	86.0	86.0	86.3	84.0	85.7	86.0	86.0	85.7	85.7	85.7	85.7	84.1	85.7	85.7	85.7	85.7	85.5	85.7	85.7	85.7	86.0	86.0	86.0	86.0	60.8	85.5	85.7	86.3	85.7	85.2	84.4	85.5	78.8	85.7	85.5	85.2	85.5	85.7	85.7	85.7	85.5	100																																		
63	P. caspiana FBF58	80.9	79.1	80.5	80.6	79.1	81.8	81.2	80.9	81.2	80.9	78.8	77.3	82.6	99.1	83.2	81.2	83.2	82.9	83.2	82.9	83.2	83.2	82.9	81.2	82.9	83.2	83.2	82.9	82.9	82.9	82.9	81.2	82.9	82.9	82.9	82.9	82.9	82.9	82.9	82.9	81.2	83.2	83.2	83.2	59.3	82.6	82.9	81.8	82.6	81.8	81.8	82.6	99.1	82.9	82.1	82.4	82.6	82.4	82.9	82.9	82.6	78.2	100																																																																												
64	Pseudomonas sp. FBF60	93.3	89.8	88.1	92.3	80.6	96.7	93.1	93.3	93.8	93.3	87.9	85.7	99.8	81.8	97.0	96.3	97.0	96.7	97.0	96.7	97.0	97.0	96.7	95.0	96.7	97.0	97.0	96.7	96.7	96.7	96.7	94.8	96.7	96.7	96.7	96.7	96.5	96.7	96.7	96.7	96.3	97.0	97.0	97.0	62	99.8	97.2	96.3	98.2	98.6	98.6	99.8	81.8	97.2	98.9	99.5	99.8	100	96.7	97.2	99.3	85.7	82.4	100																																																																										
65	Pseudomonas sp. FBF61	93.1	90.0	88.9	92.1	80.3	96.0	92.8	93.1	93.6	93.1	87.1	85.5	99.5	82.1	96.7	95.5	96.7	96.5	96.7	96.5	96.7	96.7	96.5	94.8	96.5	96.7	96.7	96.5	96.5	96.5	96.5	94.6	96.5	96.5	96.5	96.5	96.3	96.5	96.5	96.5	95.5	96.7	96.7	96.7	62	99.5	97.0	95.5	97.9	99.3	98.4	99.5	82.1	97.0	99.5	99.3	99.5	99.3	96.5	97.0	99.1	85.5	82.6	99.3	100																																																																									
66	Pseudomonas sp. FBF62	93.1	90.0	88.9	92.1	80.3	96.0	92.8	93.1	93.6	93.1	87.1	85.5	99.5	82.1	96.7	95.5	96.7	96.5	96.7	96.5	96.7	96.7	96.5	94.8	96.5	96.7	96.7	96.5	96.5	96.5	96.5	94.6	96.5	96.5	96.5	96.5	96.3	96.5	96.5	96.5	95.5	96.7	96.7	96.7	62	99.5	97.0	95.5	97.9	99.3	98.4	99.5	82.1	97.0	99.5	99.3	99.5	99.3	96.5	97.0	99.1	85.5	82.6	99.3	100	100																																																																							
67	Pseudomonas sp. FBF63	93.1	89.2	89.2	92.3	81.8	96.5	93.3	93.6	93.6	93.1	88.4	84.6	97.0	82.6	99.8	95.8	99.8	100	99.8	100	99.8	99.8	99.5	97.9	100	99.8	99.8	100	100	100	100	98.2	100	100	100	100	99.8	100	100	100	95.8	99.8	99.8	99.8	61.2	97.0	99.5	95.8	97.7	95.8	96.0	97.0	82.6	99.5	96.0	96.7	97.0	96.7	99.5	99.5	96.5	85.7	82.9	96.7	96.5	96.5	100																																																																					
68	Pseudomonas sp. FBF68	93.6	90.0	88.4	92.6	80.9	96.5	93.3	93.6	94.1	93.6	87.6	85.5	100	82.1	97.2	96.0	97.2	97.0	97.2	97.0	97.2	97.2	97.0	95.3	97.0	97.2	97.2	97.0	97.0	97.0	97.0	95.1	97.0	97.0	97.0	97.0	96.7	97.0	97.0	97.0	96.0	97.2	97.2	97.2	62	100	97.5	96.0	98.4	98.9	98.9	100	82.1	97.5	99.1	99.8	100	99.8	97.0	97.5	99.5	85.5	82.6	99.8	99.5	99.5	97.0	100																																																																				
69	Pseudomonas sp. FBF69	93.1	90.0	87.8	92.6	80.9	96.0	92.8	93.1	93.6	93.1	87.6	84.9	99.5	81.5	96.7	96.0	96.7	96.5	96.7	96.5	96.7	96.7	96.5	94.8	96.5	96.7	96.7	96.5	96.5	96.5	96.5	94.6	96.5	96.5	96.5	96.5	96.3	96.5	96.5	96.5	96.0	96.7	96.7	96.7	61.6	99.5	97.0	96.0	98.4	99.3	98.4	99.5	81.5	97.0	99.5	99.3	99.5	99.3	96.5	97.0	99.1	85.5	82.1	99.3	99.1	99.1	96.5	99.5	100																																																																			
70	Pseudomonas sp. FBF71	92.6	89.5	87.6	92.1	80.6	96.3	92.3	92.6	93.1	92.6	86.6	85.2	98.9	81.8	96.3	95.5	96.3	96.0	96.3	96.0	96.3	96.3	96.0	94.3	96.0	96.3	96.3	96.0	96.0	96.0	96.0	94.1	96.0	96.0	96.0	96.0	95.8	96.0	96.0	96.0	95.5	96.3	96.3	96.3	62.4	98.9	96.5	95.5	97.5	97.7	100	98.9	81.8	96.5	97.9	99.1	98.9	98.6	96.0	96.5	98.4	84.4	81.8	98.6	98.4	98.4	96.0	98.9	98.4	100																																																																		
71	Pseudomonas sp. FBF72	92.6	89.5	87.6	92.1	80.6	96.3	92.3	92.6	93.1	92.6	86.6	85.2	98.9	81.8	96.3	95.5	96.3	96.0	96.3	96.0	96.3	96.3	96.0	94.3	96.0	96.3	96.3	96.0	96.0	96.0	96.0	94.1	96.0	96.0	96.0	96.0	95.8	96.0	96.0	96.0	95.5	96.3	96.3	96.3	62.4	98.9	96.5	95.5	97.5	97.7	100	98.9	81.8	96.5	97.9	99.1	98.9	98.6	96.0	96.5	98.4	84.4	81.8	98.6	98.4	98.4	96.0	98.9	98.4	100	100																																																															
72	Pseudomonas sp. FBF74	92.6	89.5	87.6	92.1	80.6	96.3	92.3	92.6	93.1	92.6	86.6	85.2	98.9	81.8	96.3	95.5	96.3	96.0	96.3	96.0	96.3	96.3	96.0	94.3	96.0	96.3	96.3	96.0	96.0	96.0	96.0	94.1	96.0	96.0	96.0	96.0	95.8	96.0	96.0	96.0	95.5	96.3	96.3	96.3	62.4	98.9	96.5	95.5	97.5	97.7	100	98.9	81.8	96.5	97.9	99.1	98.9	98.6	96.0	96.5	98.4	84.4	81.8	98.6	98.4	98.4	96.0	98.9	98.4	100	100	100																																																													
73	Pseudomonas sp. FBF77	93.6	90.0	88.4	92.6	80.9	96.5	93.3	93.6	94.1	93.6	87.6	85.5	99.8	82.4	97.2	96.0	97.2	97.0	97.2	97.0	97.2	97.2	97.0	95.3	97.0	97.2	97.2	97.0	97.0	97.0	97.0	95.1	97.0	97.0	97.0	97.0	96.7	97.0	97.0	97.0	96.0	97.2	97.2	97.2	62	99.8	97.5	96.0	98.4	98.6	99.1	99.8	82.4	97.5	98.9	99.5	99.8	99.5	97.0	97.5	99.3	85.5	82.4	99.5	99.3	99.3	97.0	99.8	99.3	99.1	99.1	99.1	100																																																												
74	Pseudomonas sp. FBF78	93.6	90.0	88.4	92.6	80.9	96.5	93.3	93.6	94.1	93.6	87.6	85.5	100	82.1	97.2	96.0	97.2	97.0	97.2	97.0	97.2	97.2	97.0	95.3	97.0	97.2	97.2	97.0	97.0	97.0	97.0	95.1	97.0	97.0	97.0	97.0	96.7	97.0	97.0	97.0	96.0	97.2	97.2	97.2	62	100	97.5	96.0	98.4	98.9	98.9	100	82.1	97.5	99.1	99.8	100	99.8	97.0	97.5	99.5	85.5	82.6	99.8	99.5	99.5	97.0	100	99.5	98.9	98.9	98.9	99.8	100																																																												
75	Pseudomonas sp. FBF79	93.1	89.5	87.8	92.1	80.9	96.0	92.8	93.1	93.6	93.1	87.1	85.5	99.5	81.5	96.7	95.5	96.7	96.5	96.7	96.5	96.7	96.7	96.5	94.8	96.5	96.7	96.7	96.5	96.5	96.5	96.5	94.6	96.5	96.5	96.5	96.5	96.3	96.5	96.5	96.5	95.5	96.7	96.7	96.7	62	99.5	97.0	95.5	97.9	98.4	98.4	99.5	81.5	97.0	98.6	99.3	99.5	99.3	96.5	97.0	99.1	86.0	82.1	99.3	99.1	99.1	96.5	99.5	99.1	98.4	98.4	98.4	99.3	99.5	100																																																										
76	Pseudomonas sp. FBF82	93.6	90.0	88.4	92.6	80.9	96.5	93.3	93.6	94.1	93.6	87.6	85.5	100	82.1	97.2	96.0	97.2	97.0	97.2	97.0	97.2	97.2	97.0	95.3	97.0	97.2	97.2	97.0	97.0	97.0	97.0	95.1	97.0	97.0	97.0	97.0	96.7	97.0	97.0	97.0	96.0	97.2	97.2	97.2	62	100	97.5	96.0	98.4	98.9	98.9	100	82.1	97.5	99.1	99.8	100	99.8	97.0	97.5	99.5	85.5	82.6	99.8	99.5	99.5	97.0	100	99.5	98.9	98.9	98.9	99.8	100	99.5	100																																																										
77	Pseudomonas sp. FBF83	93.6	90.0	88.4	92.6	80.9	96.5	93.3	93.6	94.1	93.6	87.6	85.5	100	82.1	97.2	96.0	97.2	97.0	97.2	97.0	97.2	97.2	97.0	95.3	97.0	97.2	97.2	97.0	97.0	97.0	97.0	95.1	97.0	97.0	97.0	97.0	96.7	97.0	97.0	97.0	96.0	97.2	97.2	97.2	62	100	97.5	96.0	98.4	98.9	98.9	100	82.1	97.5	99.1	99.8	100	99.8	97.0	97.5	99.5	85.5	82.6	99.8	99.5	99.5	97.0	100	99.5	98.9	98.9	98.9	99.8	100	99.5	100	100																																																									
78	Pseudomonas sp. FBF91	93.3	90.3	88.1	92.8	80.6	96.7	93.1	93.3	93.8	93.3	87.4	85.7	99.8	81.8	97.0	95.8	97.0	96.7	97.0	96.7	97.0	97.0	96.7	95.0	96.7	97.0	97.0	96.7	96.7	96.7	96.7	94.8	96.7	96.7	96.7	96.7	96.5	96.7	96.7	96.7	95.8	97.0	97.0	97.0	62.4	99.8	97.2	95.8	98.2	98.6	99.1	99.8	81.8	97.2	98.9	100	99.8	99.5	96.7	97.2	99.3	85.2	82.4	99.5	99.3	99.3	96.7	99.8	99.3	99.1	99.1	99.1	99.5	99.8	99.3	99.8	99.8	100																																																							
79	Pseudomonas sp. FBF97	93.6	90.0	88.4	92.6	80.9	96.5	93.3	93.6	94.1	93.6	87.6	85.5	100	82.1	97.2	96.0	97.2	97.0	97.2	97.0	97.2	97.2	97.0	95.3	97.0	97.2	97.2	97.0	97.0	97.0	97.0	95.1	97.0	97.0	97.0	97.0	96.7	97.0	97.0	97.0	96.0	97.2	97.2	97.2	62	100	97.5	96.0	98.4	98.9	98.9	100	82.1	97.5	99.1	99.8	100	99.8	97.0	97.5	99.5	85.5	82.6	99.8	99.5	99.5	97.0	100	99.5	98.9	98.9	98.9	99.8	100	99.5	100	100	99.8	100																																																						
80	Pseudomonas sp. FBF98	92.6	89.0.5	87.6	92.1	80.6	96.3	92.3	92.6	93.1	92.6	86.6	85.2	98.9	81.8	96.3	95.5	96.3	96.0	96.3	96.0	96.3	96.3	96.0	94.3	96.0	96.3	96.3	96.0	96.0	96.0	96.0	94.1	96.0	96.0	96.0	96.0	95.8	96.0	96.0	96.0	95.5	96.3	96.3	96.3	62.4	98.9	96.5	95.5	97.5	97.7	100	98.9	81.8	96.5	97.9	99.1	98.9	98.6	96.0	96.5	98.4	84.4	81.8	98.6	98.4	98.4	96.0	98.9	98.4	100	100	100	99.1	98.9	98.4	98.9	98.9	99.1	98.9	100																																																					
81	Pseudomonas sp. FBF100	87.0	85.6	85.1	86.2	82.2	85.9	86.7	86.7	86.7	86.7	91.0	89.4	85.9	79.6	84.8	84.5	84.8	84.5	84.8	84.5	84.8	84.8	84.5	82.8	84.5	84.8	84.8	84.5	84.5	84.5	84.5	82.8	84.5	84.5	84.5	84.5	84.2	84.5	84.5	84.5	84.5	84.8	84.8	84.8	59.3	85.9	85.1	85.1	85.6	85.1	85.9	85.9	79.6	85.1	85.4	86.2	85.9	86.2	84.5	85.1	85.4	90.0	79.0	86.2	85.9	85.9	84.5	85.9	85.4	85.9	85.9	85.9	85.9	85.9	85.4	85.9	85.9	86.2	85.9	85.9	100																																																			
83	P. caspiana FBF103	80.3	79.4	81.1	80.0	79.7	81.5	80.6	80.3	80.6	80.3	79.7	77.9	82.1	99.5	83.2	80.9	83.2	82.9	83.2	82.9	83.2	83.2	82.9	81.2	82.9	83.2	83.2	82.9	82.9	82.9	82.9	81.2	82.9	82.9	82.9	82.9	82.9	82.9	82.9	82.9	80.9	83.2	83.2	83.2	59.7	82.1	82.9	81.5	82.1	81.2	81.2	82.1	99.5	82.9	81.5	81.8	82.1	81.8	82.9	82.9	82.1	78.8	99.1	81.8	82.1	82.1	82.9	82.1	81.5	81.2	81.2	81.2	81.8	82.1	81.5	82.1	82.1	81.8	82.1	81.2	79.6	100																																																		
84	Pseudomonas sp. FBF104	92.6	90.0	88.4	92.1	80.3	95.5	92.3	92.6	93.1	92.6	87.1	84.9	99.1	81.5	96.3	95.5	96.3	96.0	96.3	96.0	96.3	96.3	96.0	94.3	96.0	96.3	96.3	96.0	96.0	96.0	96.0	94.1	96.0	96.0	96.0	96.0	95.8	96.0	96.0	96.0	95.5	96.3	96.3	96.3	61.6	99.1	96.5	95.5	97.9	99.8	97.9	99.1	81.5	96.5	100	98.9	99.1	98.9	96.0	96.5	98.6	85.5	82.1	98.9	99.5	99.5	96.0	99.1	99.5	97.9	97.9	97.9	98.9	99.1	98.6	99.1	99.1	98.9	99.1	97.9	85.4	81.5	100																																																	
85	Pseudomonas sp. FBF106	93.6	90.0	88.4	92.6	80.9	96.5	93.3	93.6	94.1	93.6	87.6	85.5	100	82.1	97.2	96.0	97.2	97.0	97.2	97.0	97.2	97.2	97.0	95.3	97.0	97.2	97.2	97.0	97.0	97.0	97.0	95.1	97.0	97.0	97.0	97.0	96.7	97.0	97.0	97.0	96.0	97.2	97.2	97.2	62	100	97.5	96.0	98.4	98.9	98.9	100	82.1	97.5	99.1	99.8	100	99.8	97.0	97.5	99.5	85.5	82.6	99.8	99.5	99.5	97.0	100	99.5	98.9	98.9	98.9	99.8	100	99.5	100	100	99.8	100	98.9	85.9	82.1	99.1	100																																																
86	Pseudomonas sp. FBF107	92.6	89.5	87.6	92.1	80.6	96.3	92.3	92.6	93.1	92.6	86.6	85.2	98.9	81.8	96.3	95.5	96.3	96.0	96.3	96.0	96.3	96.3	96.0	94.3	96.0	96.3	96.3	96.0	96.0	96.0	96.0	94.1	96.0	96.0	96.0	96.0	95.8	96.0	96.0	96.0	95.5	96.3	96.3	96.3	62.4	98.9	96.5	95.5	97.5	97.7	100	98.9	81.8	96.5	97.9	99.1	98.9	98.6	96.0	96.5	98.4	84.4	81.8	98.6	98.4	98.4	96.0	98.9	98.4	100	100	100	99.1	98.9	98.4	98.9	98.9	99.1	98.9	100	85.9	81.2	97.9	98.9	100																																														
87	Pseudomonas sp. FBF108	92.6	89.5	87.6	92.1	80.6	96.3	92.3	92.6	93.1	92.6	86.6	85.2	98.9	81.8	96.3	95.5	96.3	96.0	96.3	96.0	96.3	96.3	96.0	94.3	96.0	96.3	96.3	96.0	96.0	96.0	96.0	94.1	96.0	96.0	96.0	96.0	95.8	96.0	96.0	96.0	95.5	96.3	96.3	96.3	62.4	98.9	96.5	95.5	97.5	97.7	100	98.9	81.8	96.5	97.9	99.1	98.9	98.6	96.0	96.5	98.4	84.4	81.8	98.6	98.4	98.4	96.0	98.9	98.4	100	100	100	99.1	98.9	98.4	98.9	98.9	99.1	98.9	100	85.9	81.2	97.9	98.9	100	100																																													
88	Pseudomonas sp. FBF109	93.6	91.3	88.6	93.3	80.9	97.0	93.3	93.6	94.1	93.6	87.9	85.7	97.2	82.1	96.7	96.0	96.7	96.5	96.7	96.5	96.7	96.7	96.5	94.8	96.5	96.7	96.7	96.5	96.5	96.5	96.5	94.6	96.5	96.5	96.5	96.5	96.3	96.5	96.5	96.5	96.0	96.7	96.7	96.7	62	97.2	97.0	96.0	98.4	96.5	96.7	97.2	82.1	97.0	96.7	97.5	97.2	97.0	96.5	97.0	96.7	85.7	82.4	97.0	96.7	96.7	96.5	97.2	97.2	96.7	96.7	96.7	97.2	97.2	96.7	97.2	97.2	97.5	97.2	96.7	86.2	81.8	96.7	97.2	96.7	96.7	100																																											
89	Pseudomonas sp. FBF111	93.3	89.5	89.4	92.6	82.1	96.7	93.6	93.8	93.8	93.3	88.7	84.9	97.2	82.9	100	96.0	100	99.8	100	99.8	100	100	99.8	98.2	99.8	100	100	99.8	99.8	99.8	99.8	97.9	99.8	99.8	99.8	99.8	99.5	99.8	99.8	99.8	96.0	100	100	100	61.6	97.2	99.8	96.0	97.9	96.0	96.3	97.2	82.9	99.8	96.3	97.0	97.2	97.0	99.8	99.8	96.7	86.0	83.2	97.0	96.7	96.7	99.8	97.2	96.7	96.3	96.3	96.3	97.2	97.2	96.7	97.2	97.2	97.0	97.2	96.3	84.8	83.2	96.3	97.2	96.3	96.3	96.7	100																																										
90	Pseudomonas sp. FBF113	93.1	89.5	87.8	92.1	80.9	96.0	92.8	93.1	93.6	93.1	87.1	85.5	99.5	81.5	96.7	95.5	96.7	96.5	96.7	96.5	96.7	96.7	96.5	94.8	96.5	96.7	96.7	96.5	96.5	96.5	96.5	94.6	96.5	96.5	96.5	96.5	96.3	96.5	96.5	96.5	95.5	96.7	96.7	96.7	62	99.5	97.0	95.5	97.9	98.4	98.4	99.5	81.5	97.0	98.6	99.3	99.5	99.3	96.5	97.0	99.1	86.0	82.1	99.3	99.1	99.1	96.5	99.5	99.1	98.4	98.4	98.4	99.3	99.5	100	99.5	99.5	99.3	99.5	98.4	85.4	81.5	98.6	99.5	98.4	98.4	96.7	96.7	100																																									
91	Pseudomonas sp. FBF115	93.1	89.5	87.8	92.1	80.9	96.0	92.8	93.1	93.6	93.1	87.1	85.5	99.5	81.5	96.7	95.5	96.7	96.5	96.7	96.5	96.7	96.7	96.5	94.8	96.5	96.7	96.7	96.5	96.5	96.5	96.5	94.6	96.5	96.5	96.5	96.5	96.3	96.5	96.5	96.5	95.5	96.7	96.7	96.7	62	99.5	97.0	95.5	97.9	98.4	98.4	99.5	81.5	97.0	98.6	99.3	99.5	99.3	96.5	97.0	99.1	86.0	82.1	99.3	99.1	99.1	96.5	99.5	99.1	98.4	98.4	98.4	99.3	99.5	100	99.5	99.5	99.3	99.5	98.4	85.4	81.5	98.6	99.5	98.4	98.4	96.7	96.7	100	100																																								
92	Pseudomonas sp. FBF116	93.1	89.5	87.8	92.1	80.9	96.0	92.8	93.1	93.6	93.1	87.1	85.5	99.5	81.5	96.7	95.5	96.7	96.5	96.7	96.5	96.7	96.7	96.5	94.8	96.5	96.7	96.7	96.5	96.5	96.5	96.5	94.6	96.5	96.5	96.5	96.5	96.3	96.5	96.5	96.5	95.5	96.7	96.7	96.7	62	99.5	97.0	95.5	97.9	98.4	98.4	99.5	81.5	97.0	98.6	99.3	99.5	99.3	96.5	97.0	99.1	86.0	82.1	99.3	99.1	99.1	96.5	99.5	99.1	98.4	98.4	98.4	99.3	99.5	100	99.5	99.5	99.3	99.5	98.4	85.4	81.5	98.6	99.5	98.4	98.4	96.7	96.7	100	100	100																																						
93	Pseudomonas sp. FBF117	89.8	88.2	87.8	89.0	81.8	87.1	90.3	90.3	89.8	89.8	98.2	87.9	86.8	79.4	87.4	86.8	87.4	87.1	87.4	87.1	87.4	87.4	87.6	85.4	87.1	87.4	87.4	87.1	87.1	87.1	87.1	85.5	87.1	87.1	87.1	87.1	86.8	87.1	87.1	87.1	86.8	87.4	87.4	87.4	61.6	86.8	87.1	86.6	87.1	86.6	86.3	86.8	79.4	87.1	86.8	87.1	86.8	87.1	87.1	87.1	86.3	97.7	78.8	87.1	86.8	86.8	87.1	86.8	86.8	86.3	86.3	86.3	86.8	86.8	86.8	86.8	86.8	87.1	86.8	86.3	92.0	79.4	86.8	86.8	86.3	86.3	87.6	87.4	86.8	86.8	86.8	100																																						
94	Pseudomonas sp. FBF118	92.6	89.5	87.6	92.1	80.6	96.3	92.3	92.6	93.1	92.6	86.6	85.2	98.9	81.8	96.3	95.5	96.3	96.0	96.3	96.0	96.3	96.3	96.0	94.3	96.0	96.3	96.3	96.0	96.0	96.0	96.0	94.1	96.0	96.0	96.0	96.0	95.8	96.0	96.0	96.0	95.5	96.3	96.3	96.3	62.4	98.9	96.5	95.5	97.5	97.7	100	98.9	81.8	96.5	97.9	99.1	98.9	98.6	96.0	96.5	98.4	84.4	81.8	98.6	98.4	98.4	96.0	98.9	98.4	100	100	100	99.1	98.9	98.4	98.9	98.9	99.1	98.9	100	85.9	81.2	97.9	98.9	100	100	96.7	96.3	98.4	98.4	98.4	86.3	100																																					
95	Pseudomonas sp. FBF119	92.6	90.0	88.4	92.1	80.3	95.5	92.3	92.6	93.1	92.6	87.1	84.9	99.1	81.5	96.3	95.5	96.3	96.0	96.3	96.0	96.3	96.3	96.0	94.3	96.0	96.3	96.3	96.0	96.0	96.0	96.0	94.1	96.0	96.0	96.0	96.0	95.8	96.0	96.0	96.0	95.5	96.3	96.3	96.3	61.6	99.1	96.5	95.5	97.9	99.8	97.9	99.1	81.5	96.5	100	98.9	99.1	98.9	96.0	96.5	98.6	85.5	82.1	98.9	99.5	99.5	96.0	99.1	99.5	97.9	97.9	97.9	98.9	99.1	98.6	99.1	99.1	98.9	99.1	97.9	85.4	81.5	100	99.1	97.9	97.9	96.7	96.3	98.6	98.6	98.6	86.8	97.9	100																																				
96	P. caspiana FBF122	80.6	79.4	81.6	80.3	80.3	81.5	80.9	80.6	80.9	80.6	79.4	77.9	82.1	99.3	82.9	80.9	82.9	82.6	82.9	82.6	82.9	82.9	82.6	80.9	82.6	82.9	82.9	82.6	82.6	82.6	82.6	80.9	82.6	82.6	82.6	82.6	82.6	82.6	82.6	82.6	80.9	82.9	82.9	82.9	60.1	82.1	82.6	80.9	82.4	81.2	81.8	82.1	99.3	82.6	81.5	81.8	82.1	81.8	82.6	82.6	82.1	78.8	98.9	81.8	82.1	82.1	82.6	82.1	81.5	81.8	81.8	81.8	82.4	82.1	81.5	82.1	82.1	81.8	82.1	81.8	79.6	98.9	81.5	82.1	81.8	81.8	82.1	82.9	81.5	81.5	81.5	79.4	81.8	81.5	100	100																	
97	Pseudomonas sp. FBF124	93.6	90.0	88.4	92.6	80.9	96.3	93.3	93.6	94.1	93.6	87.6	85.5	99.5	82.1	97.2	95.8	97.2	97.0	97.2	97.0	97.2	97.2	97.0	95.3	97.0	97.2	97.2	97.0	97.0	97.0	97.0	95.1	97.0	97.0	97.0	97.0	96.7	97.0	97.0	97.0	95.8	97.2	97.2	97.2	61.6	99.5	97.0	95.8	97.9	98.4	98.9	99.5	82.1	97.0	98.6	99.3	99.5	99.3	97.0	97.0	99.1	85.5	82.6	99.3	99.1	99.1	97.0	99.5	99.1	98.9	98.9	98.9	99.3	99.5	99.1	99.5	99.5	99.3	99.5	98.9	85.4	82.1	98.6	99.5	98.9	98.9	96.7	97.2	99.1	99.1	99.1	86.8	98.9	98.6	82.1	100																	
98	Pseudomonas sp. FBF125	93.6	91.3	88.6	93.3	80.9	97.0	93.3	93.6	94.1	93.6	87.9	85.7	97.2	82.1	96.7	96.0	96.7	96.5	96.7	96.5	96.7	96.7	96.5	94.8	96.5	96.7	96.7	96.5	96.5	96.5	96.5	94.6	96.5	96.5	96.5	96.5	96.3	96.5	96.5	96.5	96.0	96.7	96.7	96.7	62	97.2	97.0	96.0	98.4	96.5	96.7	97.2	82.1	97.0	96.7	97.5	97.2	97.0	96.5	97.0	96.7	85.7	82.4	97.0	96.7	96.7	96.5	97.2	97.2	96.7	96.7	96.7	97.2	97.2	96.7	97.2	97.2	97.5	97.2	96.7	86.2	81.8	96.7	97.2	96.7	96.7	100	96.7	96.7	96.7	96.7	87.6	96.7	96.7	82.1	96.7	100																
99	Pseudomonas sp. FBF134	92.6	89.5	87.6	92.1	80.6	96.3	92.3	92.6	93.1	92.6	86.6	85.2	98.9	81.8	96.3	95.5	96.3	96.0	96.3	96.0	96.3	96.3	96.0	94.3	96.0	96.3	96.3	96.0	96.0	96.0	96.0	94.1	96.0	96.0	96.0	96.0	95.8	96.0	96.0	96.0	95.5	96.3	96.3	96.3	62.4	98.9	96.5	95.5	97.5	97.7	100	98.9	81.8	96.5	97.9	99.1	98.9	98.6	96.0	96.5	98.4	84.4	81.8	98.6	98.4	98.4	96.0	98.9	98.4	100	100	100	99.1	98.9	98.4	98.9	98.9	99.1	98.9	100	85.9	81.2	97.9	98.9	100	100	96.7	96.3	98.4	98.4	98.4	86.3	100	97.9	81.8	98.9	96.7	100															
100	Pseudomonas sp. FBF135	93.6	91.3	88.6	93.3	80.9	97.0	93.3	93.6	94.1	93.6	87.9	85.7	97.2	82.1	96.7	96.0	96.7	96.5	96.7	96.5	96.7	96.7	96.5	94.8	96.5	96.7	96.7	96.5	96.5	96.5	96.5	94.6	96.5	96.5	96.5	96.5	96.3	96.5	96.5	96.5	96.0	96.7	96.7	96.7	62	97.2	97.0	96.0	98.4	96.5	96.7	97.2	82.1	97.0	96.7	97.5	97.2	97.0	96.5	97.0	96.7	85.7	82.4	97.0	96.7	96.7	96.5	97.2	97.2	96.7	96.7	96.7	97.2	97.2	96.7	97.2	97.2	97.5	97.2	96.7	86.2	81.8	96.7	97.2	96.7	96.7	100	96.7	96.7	96.7	96.7	87.6	96.7	96.7	82.1	96.7	100	96.7	100														
101	Pseudomonas sp. FBF138	93.3	89.5	89.4	92.6	82.1	96.7	93.6	93.8	93.8	93.3	88.7	84.9	97.2	82.9	100	96.0	100	99.8	100	99.8	100	100	99.8	98.2	99.8	100	100	99.8	99.8	99.8	99.8	97.9	99.8	99.8	99.8	99.8	99.5	99.8	99.8	99.8	96.0	100	100	100	61.6	97.2	99.8	96.0	97.9	96.0	96.3	97.2	82.9	99.8	96.3	97.0	97.2	97.0	99.8	99.8	96.7	86.0	83.2	97.0	96.7	96.7	99.8	97.2	96.7	96.3	96.3	96.3	97.2	97.2	96.7	97.2	97.2	97.0	97.2	96.3	84.8	83.2	96.3	97.2	96.3	96.3	96.7	100	96.7	96.7	96.7	87.4	96.3	96.3	82.9	97.2	96.7	96.3	96.7	100													
102	Pseudomonas sp. FBF139	93.6	90.0	88.4	92.6	80.9	96.5	93.3	93.6	94.1	93.6	87.6	85.5	100	82.1	97.2	96.0	97.2	97.0	97.2	97.0	97.2	97.2	97.0	95.3	97.0	97.2	97.2	97.0	97.0	97.0	97.0	95.1	97.0	97.0	97.0	97.0	96.7	97.0	97.0	97.0	96.0	97.2	97.2	97.2	62	100	97.5	96.0	98.4	98.9	98.9	100	82.1	97.5	99.1	99.8	100	99.8	97.0	97.5	99.5	85.5	82.6	99.8	99.5	99.5	97.0	100	99.5	98.9	98.9	98.9	99.8	100	99.5	100	100	99.8	100	98.9	85.9	82.1	99.1	100	98.9	98.9	97.2	97.2	99.5	99.5	99.5	86.8	98.9	99.1	82.1	99.5	97.2	98.9	97.2	97.2	100												
103	P. syringae pv. syringae IZB200	93.3	89.5	89.4	92.6	82.1	96.7	93.6	93.8	93.8	93.3	88.7	84.9	97.2	82.9	100	96.0	100	99.8	100	99.8	100	100	99.8	98.2	99.8	100	100	99.8	99.8	99.8	99.8	97.9	99.8	99.8	99.8	99.8	99.5	99.8	99.8	99.8	96.0	100	100	100	61.6	97.2	99.8	96.0	97.9	96.0	96.3	97.2	82.9	99.8	96.3	97.0	97.2	97.0	99.8	99.8	96.7	86.0	83.2	97.0	96.7	96.7	99.8	97.2	96.7	96.3	96.3	96.3	97.2	97.2	96.7	97.2	97.2	97.0	97.2	96.3	84.8	83.2	96.3	97.2	96.3	96.3	96.7	100	96.7	96.7	96.7	87.4	96.3	96.3	82.9	97.2	96.7	96.3	96.7	100	97.2	100											
104	P. syringae pv. syringae IZB201	93.3	89.5	89.4	92.6	82.1	96.7	93.6	93.8	93.8	93.3	88.7	84.9	97.2	82.9	100	96.0	100	99.8	100	99.8	100	100	99.8	98.2	99.8	100	100	99.8	99.8	99.8	99.8	97.9	99.8	99.8	99.8	99.8	99.5	99.8	99.8	99.8	96.0	100	100	100	61.6	97.2	99.8	96.0	97.9	96.0	96.3	97.2	82.9	99.8	96.3	97.0	97.2	97.0	99.8	99.8	96.7	86.0	83.2	97.0	96.7	96.7	99.8	97.2	96.7	96.3	96.3	96.3	97.2	97.2	96.7	97.2	97.2	97.0	97.2	96.3	84.8	83.2	96.3	97.2	96.3	96.3	96.7	100	96.7	96.7	96.7	87.4	96.3	96.3	82.9	97.2	96.7	96.3	96.7	100	97.2	100	100										
105	P. syringae pv. syringae IZB204	93.3	89.5	89.4	92.6	82.1	96.7	93.6	93.8	93.8	93.3	88.7	84.9	97.2	82.9	100	96.0	100	99.8	100	99.8	100	100	99.8	98.2	99.8	100	100	99.8	99.8	99.8	99.8	97.9	99.8	99.8	99.8	99.8	99.5	99.8	99.8	99.8	96.0	100	100	100	61.6	97.2	99.8	96.0	97.9	96.0	96.3	97.2	82.9	99.8	96.3	97.0	97.2	97.0	99.8	99.8	96.7	86.0	83.2	97.0	96.7	96.7	99.8	97.2	96.7	96.3	96.3	96.3	97.2	97.2	96.7	97.2	97.2	97.0	97.2	96.3	84.8	83.2	96.3	97.2	96.3	96.3	96.7	100	96.7	96.7	96.7	87.4	96.3	96.3	82.9	97.2	96.7	96.3	96.7	100	97.2	100	100	100									
106	P. syringae pv. syringae IZB208	93.3	89.5	89.4	92.6	82.1	96.7	93.6	93.8	93.8	93.3	88.7	84.9	97.2	82.9	100	96.0	100	99.8	100	99.8	100	100	99.8	98.2	99.8	100	100	99.8	99.8	99.8	99.8	97.9	99.8	99.8	99.8	99.8	99.5	99.8	99.8	99.8	96.0	100	100	100	61.6	97.2	99.8	96.0	97.9	96.0	96.3	97.2	82.9	99.8	96.3	97.0	97.2	97.0	99.8	99.8	96.7	86.0	83.2	97.0	96.7	96.7	99.8	97.2	96.7	96.3	96.3	96.3	97.2	97.2	96.7	97.2	97.2	97.0	97.2	96.3	84.8	83.2	96.3	97.2	96.3	96.3	96.7	100	96.7	96.7	96.7	87.4	96.3	96.3	82.9	97.2	96.7	96.3	96.7	100	97.2	100	100	100	100								
107	P. syringae pv. syringae IZB209	93.3	89.5	89.4	92.6	82.1	96.7	93.6	93.8	93.8	93.3	88.7	84.9	97.2	82.9	100	96.0	100	99.8	100	99.8	100	100	99.8	98.2	99.8	100	100	99.8	99.8	99.8	99.8	97.9	99.8	99.8	99.8	99.8	99.5	99.8	99.8	99.8	96.0	100	100	100	61.6	97.2	99.8	96.0	97.9	96.0	96.3	97.2	82.9	99.8	96.3	97.0	97.2	97.0	99.8	99.8	96.7	86.0	83.2	97.0	96.7	96.7	99.8	97.2	96.7	96.3	96.3	96.3	97.2	97.2	96.7	97.2	97.2	97.0	97.2	96.3	84.8	83.2	96.3	97.2	96.3	96.3	96.7	100	96.7	96.7	96.7	87.4	96.3	96.3	82.9	97.2	96.7	96.3	96.7	100	97.2	100	100	100	100	100							
108	P. syringae pv. syringae IZB212	93.3	89.5	89.4	92.6	82.1	96.7	93.6	93.8	93.8	93.3	88.7	84.9	97.2	82.9	100	96.0	100	99.8	100	99.8	100	100	99.8	98.2	99.8	100	100	99.8	99.8	99.8	99.8	97.9	99.8	99.8	99.8	99.8	99.5	99.8	99.8	99.8	96.0	100	100	100	61.6	97.2	99.8	96.0	97.9	96.0	96.3	97.2	82.9	99.8	96.3	97.0	97.2	97.0	99.8	99.8	96.7	86.0	83.2	97.0	96.7	96.7	99.8	97.2	96.7	96.3	96.3	96.3	97.2	97.2	96.7	97.2	97.2	97.0	97.2	96.3	84.8	83.2	96.3	97.2	96.3	96.3	96.7	100	96.7	96.7	96.7	87.4	96.3	96.3	82.9	97.2	96.7	96.3	96.7	100	97.2	100	100	100	100	100	100						
109	P. syringae pv. syringae IZB214	93.3	89.5	89.4	92.6	82.1	96.7	93.6	93.8	93.8	93.3	88.7	84.9	97.2	82.9	100	96.0	100	99.8	100	99.8	100	100	99.8	98.2	99.8	100	100	99.8	99.8	99.8	99.8	97.9	99.8	99.8	99.8	99.8	99.5	99.8	99.8	99.8	96.0	100	100	100	61.6	97.2	99.8	96.0	97.9	96.0	96.3	97.2	82.9	99.8	96.3	97.0	97.2	97.0	99.8	99.8	96.7	86.0	83.2	97.0	96.7	96.7	99.8	97.2	96.7	96.3	96.3	96.3	97.2	97.2	96.7	97.2	97.2	97.0	97.2	96.3	84.8	83.2	96.3	97.2	96.3	96.3	96.7	100	96.7	96.7	96.7	87.4	96.3	96.3	82.9	97.2	96.7	96.3	96.7	100	97.2	100	100	100	100	100	100	100					
110	P. syringae pv. syringae IZB218	93.3	89.5	89.4	92.6	82.1	96.7	93.6	93.8	93.8	93.3	88.7	84.9	97.2	82.9	100	96.0	100	99.8	100	99.8	100	100	99.8	98.2	99.8	100	100	99.8	99.8	99.8	99.8	97.9	99.8	99.8	99.8	99.8	99.5	99.8	99.8	99.8	96.0	100	100	100	61.6	97.2	99.8	96.0	97.9	96.0	96.3	97.2	82.9	99.8	96.3	97.0	97.2	97.0	99.8	99.8	96.7	86.0	83.2	97.0	96.7	96.7	99.8	97.2	96.7	96.3	96.3	96.3	97.2	97.2	96.7	97.2	97.2	97.0	97.2	96.3	84.8	83.2	96.3	97.2	96.3	96.3	96.7	100	96.7	96.7	96.7	87.4	96.3	96.3	82.9	97.2	96.7	96.3	96.7	100	97.2	100	100	100	100	100	100	100	100				
111	P. syringae pv. syringae IZB219	93.3	89.5	89.4	92.6	82.1	96.7	93.6	93.8	93.8	93.3	88.7	84.9	97.2	82.9	100	96.0	100	99.8	100	99.8	100	100	99.8	98.2	99.8	100	100	99.8	99.8	99.8	99.8	97.9	99.8	99.8	99.8	99.8	99.5	99.8	99.8	99.8	96.0	100	100	100	61.6	97.2	99.8	96.0	97.9	96.0	96.3	97.2	82.9	99.8	96.3	97.0	97.2	97.0	99.8	99.8	96.7	86.0	83.2	97.0	96.7	96.7	99.8	97.2	96.7	96.3	96.3	96.3	97.2	97.2	96.7	97.2	97.2	97.0	97.2	96.3	84.8	83.2	96.3	97.2	96.3	96.3	96.7	100	96.7	96.7	96.7	87.4	96.3	96.3	82.9	97.2	96.7	96.3	96.7	100	97.2	100	100	100	100	100	100	100	100	100			
112	P. syringae pv. syringae IZB221	93.3	89.5	89.4	92.6	82.1	96.7	93.6	93.8	93.8	93.3	88.7	84.9	97.2	82.9	100	96.0	100	99.8	100	99.8	100	100	99.8	98.2	99.8	100	100	99.8	99.8	99.8	99.8	97.9	99.8	99.8	99.8	99.8	99.5	99.8	99.8	99.8	96.0	100	100	100	61.6	97.2	99.8	96.0	97.9	96.0	96.3	97.2	82.9	99.8	96.3	97.0	97.2	97.0	99.8	99.8	96.7	86.0	83.2	97.0	96.7	96.7	99.8	97.2	96.7	96.3	96.3	96.3	97.2	97.2	96.7	97.2	97.2	97.0	97.2	96.3	84.8	83.2	96.3	97.2	96.3	96.3	96.7	100	96.7	96.7	96.7	87.4	96.3	96.3	82.9	97.2	96.7	96.3	96.7	100	97.2	100	100	100	100	100	100	100	100	100	100		
113	P. syringae pv. syringae IZB228	93.3	89.5	89.4	92.6	82.1	96.7	93.6	93.8	93.8	93.3	88.7	84.9	97.2	82.9	100	96.0	100	99.8	100	99.8	100	100	99.8	98.2	99.8	100	100	99.8	99.8	99.8	99.8	97.9	99.8	99.8	99.8	99.8	99.5	99.8	99.8	99.8	96.0	100	100	100	61.6	97.2	99.8	96.0	97.9	96.0	96.3	97.2	82.9	99.8	96.3	97.0	97.2	97.0	99.8	99.8	96.7	86.0	83.2	97.0	96.7	96.7	99.8	97.2	96.7	96.3	96.3	96.3	97.2	97.2	96.7	97.2	97.2	97.0	97.2	96.3	84.8	83.2	96.3	97.2	96.3	96.3	96.7	100	96.7	96.7	96.7	87.4	96.3	96.3	82.9	97.2	96.7	96.3	96.7	100	97.2	100	100	100	100	100	100	100	100	100	100	100	
114	P. syringae pv. syringae IZB231	93.3	89.5	89.4	92.6	82.1	96.7	93.6	93.8	93.8	93.3	88.7	84.9	97.2	82.9	100	96.0	100	99.8	100	99.8	100	100	99.8	98.2	99.8	100	100	99.8	99.8	99.8	99.8	97.9	99.8	99.8	99.8	99.8	99.5	99.8	99.8	99.8	96.0	100	100	100	61.6	97.2	99.8	96.0	97.9	96.0	96.3	97.2	82.9	99.8	96.3	97.0	97.2	97.0	99.8	99.8	96.7	86.0	83.2	97.0	96.7	96.7	99.8	97.2	96.7	96.3	96.3	96.3	97.2	97.2	96.7	97.2	97.2	97.0	97.2	96.3	84.8	83.2	96.3	97.2	96.3	96.3	96.7	100	96.7	96.7	96.7	87.4	96.3	96.3	82.9	97.2	96.7	96.3	96.7	100	97.2	100	100	100	100	100	100	100	100	100	100	100	100

Supplementary Fig.1 Phylogenetic tree built with Neighbor joining method based on *cts* partial sequences of *P. syringae* strains isolated from Tunisian citrus orchards

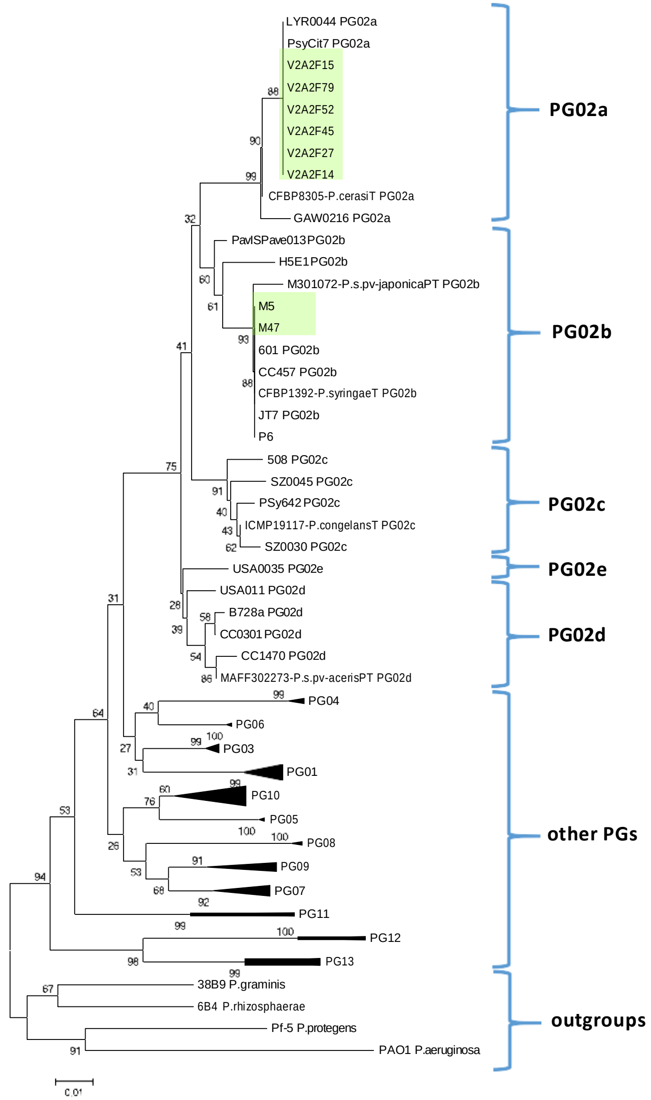

Supplementary Fig.2 BOX fingerprints of representative strains of the *P. syringae* group isolated from citrus in symptomatic Tunisian orchards from different regions

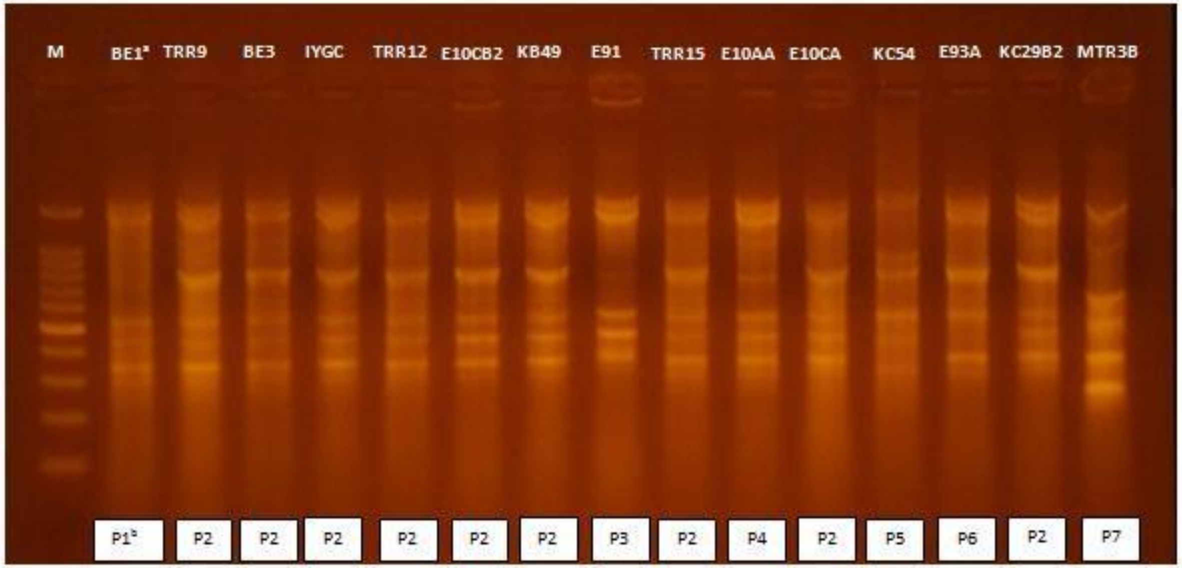

[a] Strain names

[b] Profile number: two strains sharing the same Box profile are considered closely related and belonging the same species/phylogroup

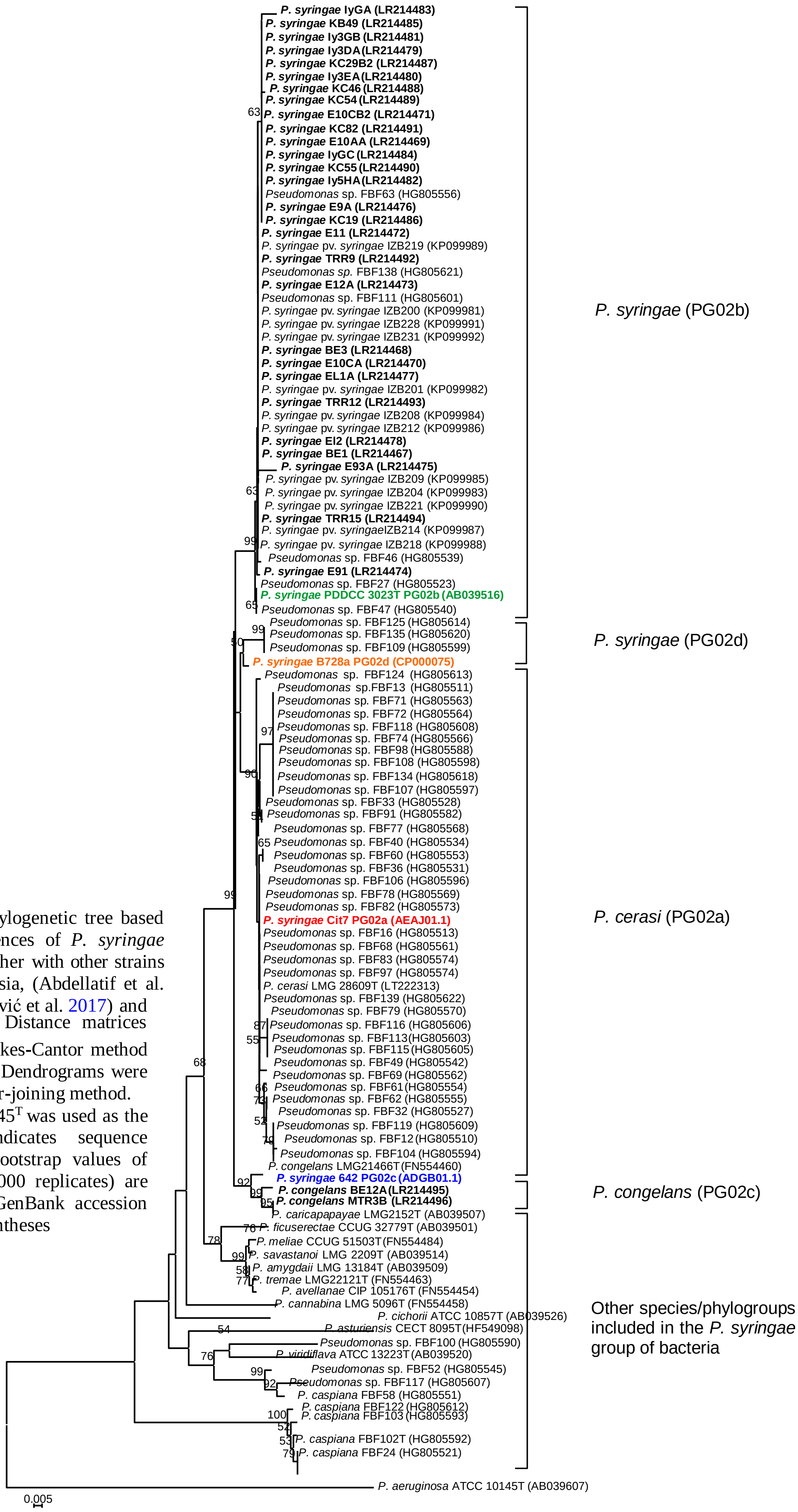

Supplementary Fig.3 Phylogenetic tree based on the *rpoD* gene sequences of *P. syringae* strains of this study, together with other strains of *P. syringae* from Tunisia, (Abdellatif et al. 2017), Montenegro (Ivanović et al. 2017) and Iran (Beiki et al. 2016). Distance matrices were calculated by the Jukes-Cantor method (Jukes and Cantor 1969). Dendrograms were generated by the neighbour-joining method. *P. aeruginosa* ATCC 10145^T was used as the outgroup. The bar indicates sequence divergence. Percentage bootstrap values of more than 50% (from 1000 replicates) are indicated at the nodes. GenBank accession numbers are given in parentheses
